# Integration of multi-omics and single-cell transcriptome reveals mitochondrial outer membrane protein-2 (MTX-2) as a prognostic biomarker and characterizes ubiquinone metabolism in lung adenocarcinoma

**DOI:** 10.7150/jca.106902

**Published:** 2025-04-13

**Authors:** Sachin Kumar, Chung-Che Wu, Fitria Sari Wulandari, Chung-Chieh Chiao, Ching-Chung Ko, Hung-Yun Lin, Juan Lorell Ngadio, Cathleen Rebecca, Do Thi Minh Xuan, Dahlak Daniel Solomon, Michael Michael, Lidya Kristiani, Jian-Ying Chuang, Ming-Cheng Tsai, Chih-Yang Wang

**Affiliations:** 1 Graduate Institute of Cancer Biology and Drug Discovery, College of Medical Science and Technology, Taipei Medical University, Taipei 11031, Taiwan.; 2 Faculty of Applied Sciences and Biotechnology, Shoolini University of Biotechnology and Management Sciences, Himachal Pradesh, 173229, India.; 3 Department of Neurosurgery, Taipei Medical University Hospital, Taipei, Taiwan.; 4 Department of Surgery, School of Medicine, College of Medicine, Taipei Medical University, Taipei, Taiwan.; 5 Department of Surgery, Division of Neurosurgery, Shuang Ho Hospital, Taipei Medical University, New Taipei City, Taiwan.; 6 Ph.D. Program for Cancer Molecular Biology and Drug Discovery, College of Medical Science and Technology, Taipei Medical University, Taipei 11031, Taiwan; 7 Department of Medical Imaging, Chi-Mei Medical Center, Tainan, Taiwan.; 8 Department of Health and Nutrition, Chia Nan University of Pharmacy and Science, Tainan, Taiwan.; 9 School of Medicine, College of Medicine, National Sun Yat-Sen University, Kaohsiung, Taiwan.; 10 Cancer Center, Wan Fang Hospital, Taipei Medical University, Taipei 11031, Taiwan.; 11 TMU Research Center of Cancer Translational Medicine, Taipei Medical University, Taipei 11031, Taiwan.; 12 Traditional Herbal Medicine Research Center of Taipei Medical University Hospital, Taipei Medical University, Taipei 11031, Taiwan.; 13 Pharmaceutical Research Institute, Albany College of Pharmacy and Health Sciences, Rensselaer, NY 12144, USA.; 14 Department of Bioinformatics, School of Life Sciences, Indonesia International Institute for Life Sciences, Jl. Pulomas Barat Kav 88, Jakarta Timur, 13210, Indonesia.; 15 Department of Pharmacy, School of Medicine and Health Sciences, Atma Jaya Catholic University of Indonesia, Jakarta, Indonesia.; 16 Faculty of Pharmacy, Van Lang University, 69/68 Dang Thuy Tram Street, Ward 13, Binh Thanh District, Ho Chi Minh City 70000, Vietnam. Electronic address: xuan.dtm@vlu.edu.vn.; 17 Yogananda School of AI Computers and Data Sciences, Shoolini University, Solan 173229, India.; 18 Department of Biomedicine, School of Life Sciences, Indonesia International Institute for Life Sciences, Jl Pulomas Barat Kav 88, Jakarta Timur, 13210, Indonesia.; 19 The Ph.D. Program for Neural Regenerative Medicine, College of Medical Science and Technology, Taipei Medical University, Taipei 11031, Taiwan.; 20 Cell Physiology and Molecular Image Research Center, Wan Fang Hospital, Taipei Medical University, Taipei 11031, Taiwan.; 21 Department of Biomedical Science and Environmental Biology, Kaohsiung Medical University, Kaohsiung 80708, Taiwan.; 22 School of Medicine, Fu Jen Catholic University, New Taipei City 242, Taiwan.; 23 Department of Neurosurgery, Shin-Kong Wu Ho-Su Memorial Hospital, 95 Wen-Chang Road, Shih-Lin District, Taipei 111045, Taiwan.

**Keywords:** Metaxin-2 (MTX2), Lung Adenocarcinoma (LUAD), Immune Infiltration, Cell Metabolism, Bioinformatics.

## Abstract

Lung adenocarcinoma (LUAD) remains to be one of the most prevalent and highly invasive forms of cancer. Mitochondrial outer membrane protein-2 or Metaxin-2 (MTX2), a key regulator of mitochondrial function, has been linked to cellular bioenergetics and stress response mechanisms. However, its roles in the progression and prognosis of LUAD remain largely unexplored. This study, employed a multi-omics approach, integrating transcriptomic and clinical patient data from public databases, to evaluate the expression and prognostic relevance of MTX2 in LUAD. Single-cell RNA sequencing was utilized to further explore MTX2's role in immune infiltration and interactions within the tumor microenvironment. Additionally, we validated these findings through a series of molecular biology and functional assays. Our results demonstrated that MTX2 expression was higher in LUAD tissues compared to normal lung tissues. Elevated MTX2 levels were significantly associated with poorer overall survival in LUAD patients. Functional analyses revealed that MTX2 regulates mitochondrial bioenergetics and facilitates tumor cell proliferation. Additionally, MTX2 expression was associated with increased immune cell infiltration. A pathway analysis identified cell metabolic and tumor growth pathways regulated by MTX2, supporting its role in tumor progression. Our research identifies MTX2 as a promising prognostic biomarker and therapeutic target for LUAD. Increased expression of MTX2 promotes tumor growth by altering metabolic pathways and modulating the immune response, underscoring its potential as a new target for LUAD treatment.

## Introduction

Lung adenocarcinoma (LUAD) is one of the most prevalent and lethal forms of lung cancer globally, constituting a leading cause of cancer-related mortality. It accounts for approximately 40% of all diagnosed lung cancer cases^1^. Lung adenocarcinoma (LUAD) originates from type II alveolar epithelial cells. These cells play a crucial role in the lungs by producing mucus and other substances that help maintain the respiratory tract's health and function^2^, ^3^. This disease is characterized by the formation of malignant neoplasms in the lungs, leading to significant global morbidity and mortality due to its aggressive progression, late-stage diagnosis, and limited treatment options^4^. The effectiveness of standard treatment modalities, including surgery, radiation therapy, and chemotherapy, is predicated on their ability to target specific molecules involved in the initiation, progression, and metastasis of cancers^5^. Molecular-targeted therapies provide the benefit of precise drug delivery with minimal off-target effects compared to traditional chemotherapy. Therefore, it is crucial to identify optimal therapeutic targets and novel biomarkers for early diagnosis and improved prognosis, as well as to develop personalized molecular therapies for treating malignancies.^6^.

The risk of developing lung cancer indeed increases with age, as the disease is less common in people below the age of 50 years^7^. Moreover, men are more susceptible to lung cancer than are women. Interestingly, while smoking accounts for 80%-90% of lung cancer cases, only approximately 15% of smokers are affected, suggesting a genetic predisposition^8^. Smoking intensity and the cumulative duration of smoking equally influence the risk^9^. Therefore, quitting smoking significantly reduces the risk of lung cancer^10^. Additional risk factors include passive smoking, cigar and pipe smoking, and potentially marijuana, but the evidence remains unclear due to confounding factors and limited long-term data^11^. There is a significant synergistic effect between asbestos exposure and tobacco use, leading to a higher incidence of lung cancer compared to either risk factor alone. Furthermore, a history of chronic obstructive pulmonary disease (COPD) and a family history of lung cancer are also linked with an increased risk of developing the disease, even when accounting for cigarette smoking.^12^. Despite significant progress in understanding the molecular mechanisms of LUAD, there remains an urgent need to identify new biomarkers and therapeutic targets to enhance patient outcomes.

**Figure 1:**
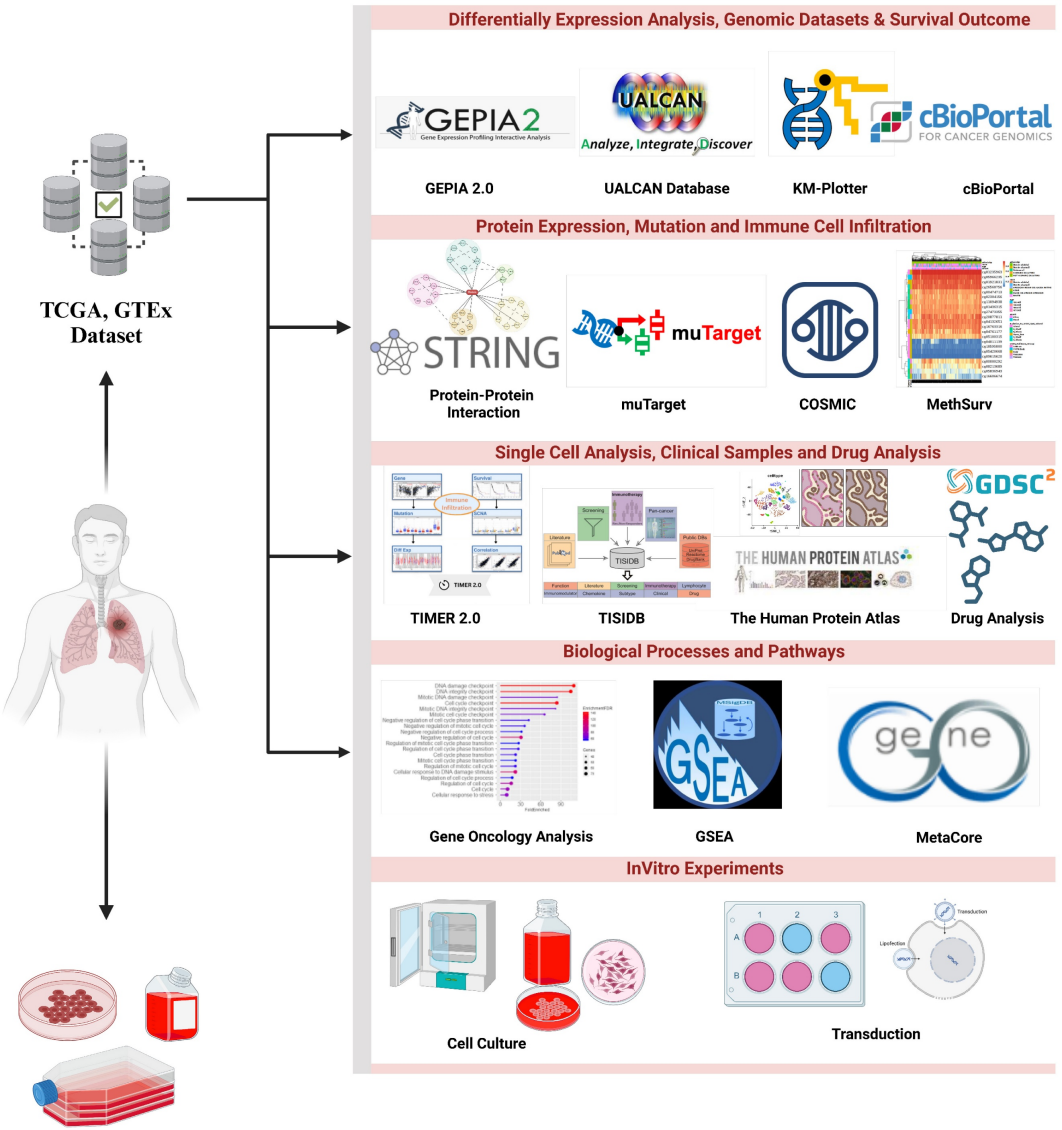
** Flowchart of the study design and analytical steps in the present study on lung adenocarcinoma.** Gene data were collected from publicly available databases, i.e., TCGA (Xena Browser), to investigate gene expression levels in lung cancer tissues compared to normal lung tissues and their putative connection with patient prognoses.

The metaxin-2 (*MTX2*) gene, located at chromosome 2q31.1 (Supplementary Fig. S1A) spans 68,631 bases and encodes 263 amino acids for mitochondrial outer membrane import complex protein 2, with a molecular weight of 29.763 kDa (Supplementary Fig. S1B). Metaxin-2 interacts with metaxin-1 (MTX1) and is involved in protein transportation into mitochondria. MTX2 is associated with the mitochondrial contact site and crista organizing system (MICOS) complex, which includes MICOS10/MIC10, CHCHD3/MIC19, CHCHD6/MIC25, APOOL/MIC27, IMMT/MIC60, APOO/MIC23/MIC26, and QIL1/MIC13, formerly known as the MINOS/MitOS complex. In this study, we investigated the significance of the *MTX2* gene in LUAD, by exploring its biological roles, dysregulation in cancer, and potential as a diagnostic, prognostic, and therapeutic target. This study represents the first attempt to conduct a statistical analysis of MTX2 in lung cancer patients by harnessing various comprehensive internet databases. A flowchart is depicted in Fig. 1 that outlines the research methodologies employed in this investigation, including an expression level analysis, clinical survival, and functional enrichment related to MTX2 in lung cancer.

## Materials and Methods

### Data collection

Gene profiles and clinical survival data of LUAD patient samples were retrieved from The Cancer Genome Atlas (TCGA) database using the UCSC Xena browser (https://xena.ucsc.edu/)^13^. Raw data were obtained from TCGA, comprising 11,069 samples from 33 types of cancer. The collected data and expression levels were assessed between cancer samples and matched reference samples. The expression data was Log2-transformed, and differential expression between cancerous and normal tissues was evaluated using two separate t-tests, with statistical significance defined as p < 0.05. Further validation using two different datasets was performed, one for survival and one for a bulk-RNA analysis. For the survival analysis, the Director's Challenge Consortium for the Molecular Classification of Lung Adenocarcinoma from GSE68465 was used^14^, while RNA-sequencing (RNA-Seq) used GSE81089 dataset^15^. The former consisted of microarray data from 343 patients with complete clinical data, while the latter consisted of RNA-Seq data from LC data compared to normal samples. These analyses provide crucial insights into the molecular mechanisms of LUAD and emphasize the potential of MTX2 as a biomarker or therapeutic target for this aggressive malignancy.

### *MTX2* gene expression analysis

GEPIA 2.0 (http://gepia2.cancer-pku.cn/index.html) is a web platform that utilizes RNA-Seq expression data from 9736 malignancies and 8587 normal samples from TCGA and GTEx projects. An independent *t*-test was conducted to determine *p* values*,* and statistical significance was defined at *p* < 0.05^16^. UALCAN (http://ualcan.path.uab.edu/analysis.html) is a user-friendly web resource designed to facilitate the analysis of cancer omics data. It provides tools for biomarker identification, graphical visualization of gene expression and patient survival data, epigenetic regulation evaluations, and a pan-cancer gene expression analysis. The platform also integrates data from external databases, facilitating clinical proteomic consortium data analyses and offers insights into LUAD-related gene and protein expressions, making it a valuable resource for researchers seeking in-depth insights into genes of interest in the context of cancer research^17^. Omics Playground vers. 3.4.1^18^ and SRPlot^19^ are online statistical analytical tools that store a collection of data from the gene expression omnibus (GEO). These tools were used to analyze RNA-Seq data from GSE81089. Data filtering was done to select the adenocarcinoma histological subtype in the dataset. DESeq2 was then utilized to produce a list of differentially expressed genes (DEGs) and was visualized in a volcano plot with a cutoff Log (fold change [FC]) of 0.5 and a false detection rate (FDR) of 0.5. MTX2 data were then plotted as a boxplot to make more explicit comparisons between tumor and normal patients.

### Immune infiltration and survival analysis of MTX2 expression

TIMER (https://cistrome.shinyapps.io/timer/) is an integrated computing tool designed to analyze and visualize genetic data and tumor immunology^20^. In this study, TIMER and EPIC algorithms were utilized to assess associations between gene expressions and immune infiltration using Spearman's test, with results displayed as scatterplots. TISIDB (http://cis.hku.hk/TISIDB/index.php), a platform integrating heterogenous data types, was used to explore interactions between tumors and the immune system^21^. For this research, the analysis followed a practice of using two-sided *p* values with a specific significance threshold of *p* < 0.05. The study utilized TIMER to evaluate relationships between MTX2 and various types of immune cell infiltration, including B cells, cluster of differentiation 4-positive (CD4^+^) and CD8^+^ T cells, neutrophils, macrophages, and dendritic cells, across different malignancies.

The Kaplan-Meier (KM) Plotter is a widely utilized approach for estimating survival times and probabilities. It provides a tool for assessing the impact of 54,000 genes on survival outcomes across 21 distinct cancer types, such as breast, ovarian, lung, and gastric cancers. These databases aggregate data from various sources, including GEO and TCGA^22^. In this study, the KM Plotter was used to investigate the prognostic significance of messenger (m)RNA expression of *MTX2* in LUAD. Patients were stratified into high- and low-expression groups according to the median MTX2 expression level, and survival differences between the groups were assessed using a log-rank test, with statistical significance defined as p < 0. 05. Univariate and multivariate Cox analyses were performed in R using the "survival" package with data from GSE68465. Clinical data were pre-processed by categorizing cancer stages into low (stages I and II) and high stages (III and IV) and consolidating the smoking status into "ever smoked" and "never smoked." Reference groups for the analyses were defined as low-stage for cancer stage, never smoked for the smoking status, and poorly differentiated for the tumor grade. Results of the Cox analyses were visualized in a single forest plot to enhance clarity and interpretation.

### COSMIC and MuTarget platform

COSMIC (https://cancer.sanger.ac.uk/cosmic/), a high-resolution database for investigating genetic targets and patterns in human cancer, discloses details regarding coding and non-coding mutations, gene fusions, genome rearrangements, abnormal copy number segments, unusual expression variants, and differentially methylated CpG dinucleotides. This comprehensive database integrates whole-genome sequencing findings from tumors and various publications, making it the most extensive repository of cancer mutations worldwide at present^23^. The MuTarget platform (https://www.mutarget.com/) was utilized to examine different mutant genes linked to alterations in MTX2 expression across various cancer types, using default thresholds. The database primarily underscores interactions among target proteins of multifunctional drugs. These targets are sorted based on their molecular functions and pathways^24^.

### Analysis of MTX2 protein expression, clinical samples, and single-cell analysis

The Search Tool for the Retrieval of Interacting Genes/Proteins (STRING) database (https://string-db.org/) is a globally recognized resource for acquiring, integrating, and evaluating published protein-protein interaction (PPI) data. It also enhances the information through computations, predictions, and analyses. With data available for over 10 different species, it contains an impressive 19,303 protein links specifically for humans^25^. The dataset utilized in this study originated from published work^26^. To better understand the tumor microenvironment (TME), a single-cell (sc)RNA-Seq analysis was conducted using a processed version of the data, which included only lung tissue samples. The original dataset, available in Hierarchical Data Format version 5 (h5ad), was converted to R Data Serialization (rds) format using the "convert Format" method from the sceasy package (vers. 0.0.7), as the analysis was performed in R Studio^27^. To further analyze the data, the converted file was imported into Seurat (version 5.1.0)^28^. The Single-Cell Sequencing Pipeline (SCP) R package (version 0.5.6) was employed to perform the scRNA-Seq analysis, enabling systematic dimensionality reduction, clustering, and data pre-processing^29^-^31^. This dataset offers a high-resolution view of the lung tissue environment, encompassing 58,870 genes across 35,685 cells. Visualization and detailed heatmap analyses were performed using the Complex Heatmap package (version 2.22.0)^28^, facilitating the identification of significant patterns and relationships within the data as we previously described^32^-^34^.

### cBioPortal and DNA methylation analysis

cBioPortal (https://www.cbioportal.org/) was developed by leading cancer research institutions to explore and analyze extensive cancer genomics data. This platform enables an in-depth analysis of complex cancer-omics datasets, serving as a valuable resource for researchers^35^. We utilized the MethSurv database (https://biit.cs.ut.ee/methsurv/) to analyze single CpG methylation patterns and generate a heatmap of distinct DNA methylated regions. DNA methylation values are represented as beta values, which fall within the range of 0-1. To compute the methylation level at each CpG site, the formula M / (M + U + 100) was employed, with 'M' and 'U' respectively denoting intensity values of methylated and unmethylated DNA^36^.

### Drug analysis and functional enrichment analysis

The GSCA database (http://bioinfo.life.hust.edu.cn/GSCA/#/drug) was utilized to collect information to determine the drug sensitivity results of a gene^37^. The GSCA database combines gene expressions from TCGA and conducts drug analysis interactions based on prior research according to the linked genes input to the database. Results are collected based on target genes searched for in the database, and gene-associated drug susceptibility can be determined.

Functional annotation and enrichment analyses are fundamental in bioinformatics for assigning biological significance to gene sequences. This study utilized gene ontology (GO) and the Kyoto Encyclopedia of Genes and Genomes (KEGG). GO provides a structured framework to classify gene functions into three categories: cellular components (CCs), molecular functions (MFs), and biological processes (BPs). On the other hand, KEGG offers a detailed resource for analyzing gene functions and mapping their biological pathways through databases, such as GENES, PATHWAY, and LIGAND. To conduct the analysis, researchers utilized cBioPortal which aggregates datasets across various categories, including gene correlations and co-expression patterns, by leveraging mRNA expression data. A functional annotation analysis was performed using Shiny GO (http://bioinformatics.sdstate.edu/go/), which enables researchers to delve into BPs, MFs, CCs, and KEGG enrichment analyses, utilizing established methodologies and tools^38^-^40^. A pathway enrichment analysis was first performed for a differential analysis. Subsequently, the Hallmark database was utilized to conduct gene set enrichment analysis (GSEA) for pathway characterization. The degree of statistical GSEA with significance is indicated by *p* values, while a normalized enrichment score (NES) reveals the rank of gene classes^41^. Afterward, MetaCore (https://portal.genego.com) was used to explore biomarker networks and cell signaling pathways, as we previous described^42^, ^43^.

### Cell culture and RT-qPCR

The A549 human lung alveolar type II epithelial cell line, generously provided by Prof. Chiou-Feng Lin of Taipei Medical University^44^-^46^, was cultured in Dulbecco's modified Eagle medium (DMEM) (90-113-PB, Corning International, Taipei City, Taiwan) with 10% fetal bovine serum (Avantor, Singapore) and 1% penicillin/streptomycin (Corning). The cells were maintained at 37°C in a humidified incubator with 5% CO2. The short hairpin (sh)RNA sequence targeting the *MTX2* gene was purchased from the National RNAi Core Facility, Academia Sinica (Taipei City, Taiwan). HEK-293T cells were transfected with the shRNA plasmid and packaging plasmid following the manufacturer's instructions. Transfected cells were cultured until lentiviral particles were produced. Supernatants containing lentiviruses were collected, filtered, and concentrated. A549 cells were transduced with lentiviruses in the presence of polybrene (8 μg/mL). Puromycin (2 μg/mL; Cyrusbio, Taiwan) was used to establish stable clones. Non-target control (pLKO.1) shRNA against luciferase was employed as an expression control.

Total RNAs was isolated using the GENEzol™ TriRNA Pure Kit (Geneaid Biotech, Xintaiwu Road, New Taipei City, Taiwan) following the manufacturer's protocol, whereas complementary (c)DNA was subsequently reverse-transcribed using a PrimeScript Synthesis Kit (Takara Bio, Taipei City, Taiwan). RT-qPCR was conducted using cDNAs as templates and Takara TB Green® SYBR green (Takara Bio, Taipei City, Taiwan) on a Roche^tm^ Light Cycler 96 platform (Roche Taiwan Pharmaceuticals, Taipei City, Taiwan). Primer pair sequences targeting *MTX2*, *CoQ10A*, and *CoQ10B*, were constructed by OriGene Technologies, Inc. (Rockville, USA). Relative fold changes (FCs) in expression of the *MTX2*, *CoQ10A*, and *CoQ10B* genes were calculated by the 2-∆∆Ct method after being normalized against the Ct value of GAPDH as an internal control. All experiments were performed in triplicate. RT-qPCR results are presented as the mean ± standard deviation (SD)^47^-^51^.

### Western blot and cell proliferation assays

Cells were lysed with 1× RIPA lysis buffer (Cyrusbio, New Taipei City, Taiwan) containing a 1% phosphatase and protease inhibitor cocktail for 30 min on ice. Lysate samples were centrifuged at 15,000 *g* for 20 min, and the supernatant was collected. Protein concentrations were measured with a BCA Protein Assay Kit (Omics Bio, New Taipei City, Taiwan). Protein samples were separated by 10% sodium dodecylsulfate polyacrylamide gel electrophoresis (SDS-PAGE) and were then transferred to a polyvinylidene fluoride membrane for 100 min at 100 V. The membrane was blocked with 5% skimmed milk for 1 h at room temperature, followed by washing with TBST thrice and incubated with antibodies at 4 ℃ overnight. After discarding the primary antibodies, the membranes were incubated with the secondary antibody at room temperature for 2 h. Blot signals were developed with an ECL substrate reagent kit (Thermo Scientific™., Taipei City, Taiwan) on an e-BLOT Touch Imager (e-BLOT, Shanghai, China.). The following antibodies were used: MTX2 (AbClonal, cat. no. A24210; Taipei City, Taiwan); GAPDH (Sigma-Aldrich cat. no. AB2302; St. Louis, MO, USA); goat anti-mouse immunoglobulin G (IgG) antibody (Sigma-Aldrich cat. no: AP124P); and goat anti-rabbit IgG antibody (Sigma-Aldrich cat. no. AP132).

Cell viability was evaluated using a 3-(4, 5-dimethylthiazolyl-2)-2, 5-diphenyltetrazolium bromide (MTT) assay to measure A549 cell proliferation. Cells were seeded in 96-well plates at 5×10^3^ cells/well in three independent experiments. The MTT solution was added to each well at a final concentration of 0.5 mg/mL and incubated at 37°C for 4 hours. Following incubation, the supernatant was removed, and the resulting formazan crystals were solubilized in 200 μL of DMSO (Cyrusbio). The absorbance was then measured at an optical density (OD) of 570 nm.

### Colony-formation and wound-healing assays

A549 cells were seeded at a density of 10³ cells per well in 6-well plates and cultured for 7 days until macroscopic colonies developed. After the incubation period, the medium was removed, and the cells were fixed with absolute ethanol at room temperature for 20 minutes. The fixed cells were then stained with a 2% methylene blue solution for 30 minutes at room temperature. Colony formation was quantified using a low-magnification light microscope^52^-^55^. The wound-healing assay, cells were seeded in six-well plates and cultured until they reached 90% confluence. A 10-μL pipette tip was used to create a scratch, followed by washing with phosphate-buffered saline (PBS) to remove detached cells. Cell migration was assessed by measuring the wound closure at 0- and 24-hours post-scratch, and images were captured using an Olympus IX73 microscope (Taichung, Taiwan).

### Statistical analysis

The bioinformatics analyses in this study were conducted using online databases. Data visualization and statistical analyses were performed with ggplot2, SPSS (IBM, Armonk, NY, USA), and ImageJ (National Institutes of Health, Bethesda, MD, USA). Results are presented as the mean ± SD, derived from a minimum of three independent experiments. Statistical analyses were carried out using one- and two-way analysis of variance (ANOVA). Survival analyses were conducted using the Kaplan-Meier method, with differences assessed by the log-rank test. The Bonferroni method was employed to determine the significance value and p-value. Differences between groups were considered significant at p<0.05, as previously described^56^-^58^.

## Results

### Expression level of *MTX2* in LUAD and survival analysis

**Figure 2:**
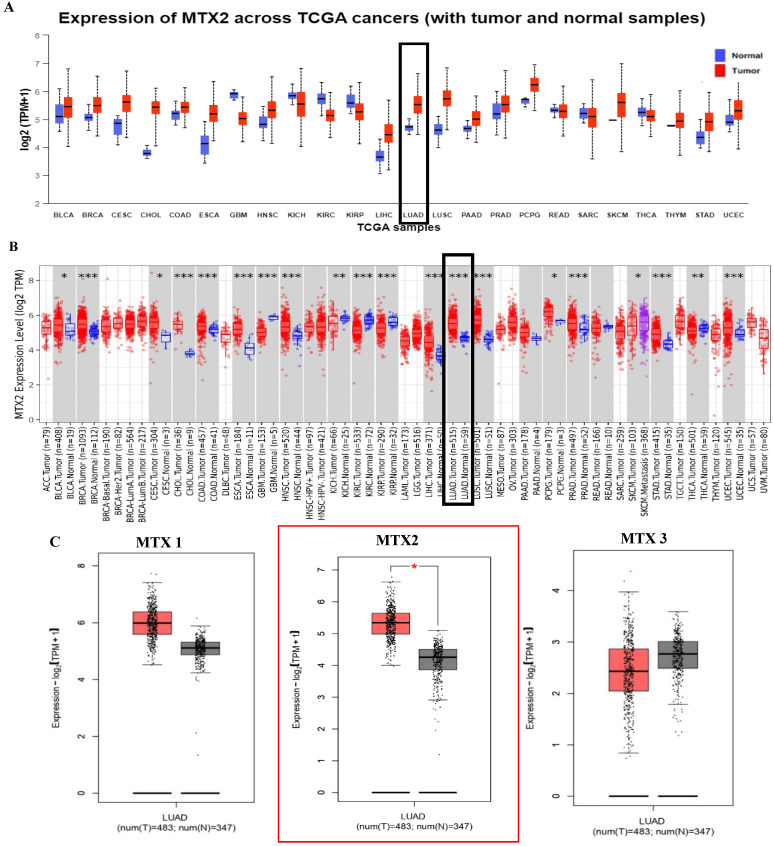
**Increased *MTX2* gene expression in lung adenocarcinoma (LUAD) patient tissues compared to normal tissues. A**: UALCAN was used to analyze *MTX2* gene expression across TCGA samples, comparing normal (blue) and tumor (red) samples. **B:** It focused on *MTX2* gene differential expressions between tumor and adjacent normal tissues across all TCGA tumors, using box plots with significance (* p < 0.05; ** p < 0.01; *** p < 0.001) to identify up- or downregulated genes in tumors for specific cancer types. **C.** The GEPIA tool was employed to assess expression levels of the *MTX2* gene in TCGA LUAD tumors (*n*=483) and TCGA normal samples (*n*=347), with data analyzed using a one-way ANOVA.

**Figure 3:**
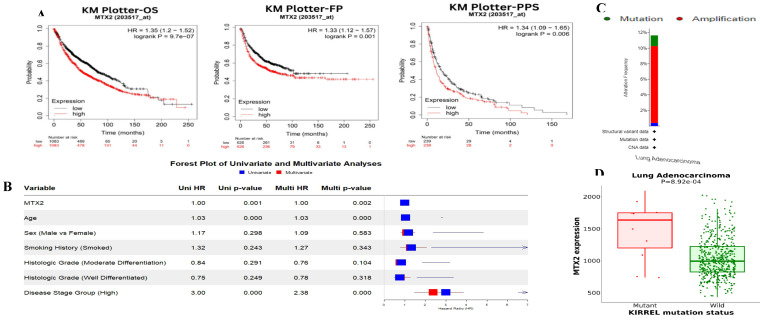
** Survival analysis, *MTX2* gene expression and genomic alterations of MTX2 in lung adenocarcinoma (LUAD): A:** Overall survival (OS), first-progression survival (FPS), and post-progression survival (PPS) curves of patients with LUAD depicting higher levels of *MTX2* mRNA linked to improved OS (*p* = 9.7e-07), FPS (*p* = 0.001), and PPS (*p* = 0.006) in both groups. However, higher expression of *MTX2* mRNA was significantly associated with reduced OS (*p* = 9.7e-07).** B:** Univariate (blue) and multivariate (red) Cox survival analysis using the survival package in R. The dataset was taken from Director's Challenge Consortium for the Molecular Classification of Lung Adenocarcinoma (GSE68465). **C:** Graphical representation of genomic alterations in the *MTX2* gene across the sample set was generated. Red bars denote gene amplifications, while blue bars represent deep deletions for each gene-tumor combination. **D:** Increased expression levels were observed in mutated KIRREL compared to wild-type KIRREL.

In this study, we examined expression levels of the *MTX2* gene family in LUAD. Based on TCGA and GTEx datasets, out of 24 cancer types studied, MTX2 was significantly upregulated in 17 types compared to normal tissues (Fig. 2A). The TIMER analysis revealed that MTX2 was significantly upregulated (*p* < 0.05 to *p* < 0.001) in multiple cancers, including LUAD, (Fig. 2B). Using GEPIA 2.0, we generated box plots to analyze expression levels of *MTX* gene family members in LUAD. Among these, *MTX2* exhibited significantly higher mRNA expression in LUAD tissues compared to normal tissues. Moreover, *MTX2* showed the highest expression levels in LUAD compared to other cancer types (Fig. 2C). This observation was further validated using NCBI GEO data (GSE81089), which confirmed a substantial increase in MTX2 expression in cancer tissues (*p* = 2.79 × 10⁻⁷, FDR = 3.01 × 10⁻⁶; (Supplementary Fig. 2A, B). To assess the prognostic significance of MTX2 expression levels in LUAD patients, we utilized the KM Plotter database to analyze overall survival (OS), first-progression survival (FPS), and post-progression survival (PPS) (Fig. 3A). The analysis identified a significant correlation between elevated MTX2 expression and reduced survival outcome. Specifically, patients with elevated MTX2 expression demonstrated significantly reduced OS with a hazard ratio of 1.35 (95% confidence interval (CI): 1.2-1.52; *p* < 0.01). These findings aligned with prior studies emphasizing the prognostic value of gene expression profiles in LUAD outcomes^59^, ^60^. This was further supported by patient-derived data which showed that MTX2 expression was highly correlated with patient survival (Fig. 3B). The clinical relevance of MTX2 as a prognostic biomarker was highlighted in a study where LUAD patients with high MTX2 expression exhibited significantly lower OS rates compared to those with low expression levels^61^. This underscores the potential utility of integrating an MTX2 expression analysis into clinical practice to improve risk stratification and treatment decision-making. Moreover, our investigation into genetic alterations associated with MTX2 utilized data from cBioPortal. Among 503 LUAD samples analyzed from the Pan-Cancer Atlas, an alteration frequency of 11.53% was observed; specifically, mutations were present in 1.39% of cases and amplifications in 9.74% (Fig 3C). These genetic changes not only reinforce the potential role of MTX2 as a key player in LUAD pathogenesis but also suggest its involvement in tumor aggressiveness and response to therapies^62^.

### Mutation analysis of MTX2 in LUAD

The MuTarget analysis revealed that mutations in KIRREL were associated with altered *MTX2* expression in LUAD (Fig. 3D). Furthermore, COSMIC data illustrated various mutation types: 1.23% (six of 486 samples) for nonsense substitutions, 15.64% (76 of 486 samples) for missense substitutions, and 4.32% (21 of 486 samples) for synonymous substitutions. Notably, in-frame insertions were absent (0%, 0 of 486 samples), while frameshift insertions causing alterations in the reading frame constituted 0.21% (one of 486 samples). However, there were no instances of in-frame deletions (0%) or frameshift deletions (0%) in this dataset. Complex mutations were not observed (0%). Specific base pair mutations identified among LUAD samples in the *MTX2* gene included: A > C six of 101 (5.94%), A > G 11 of 101 (10.89%), A > T two of 101 (1.98%), C > A eight of 101 (7.92%), C > T 21 of 101 (20.79%), C > G four of 101 (3.96%), G > A 20 of 101 (19.80%), G > C two of 101 (1.98%), G > T 12 of 101 (11.88%), T > A five of 101 (4.95%), T > C five of 101 (4.95%), and T > G five of 101 (4.95%) (Supplementary Fig. S3A, B).

### Differential expression of MTX2 in UALCAN

**Figure 4:**
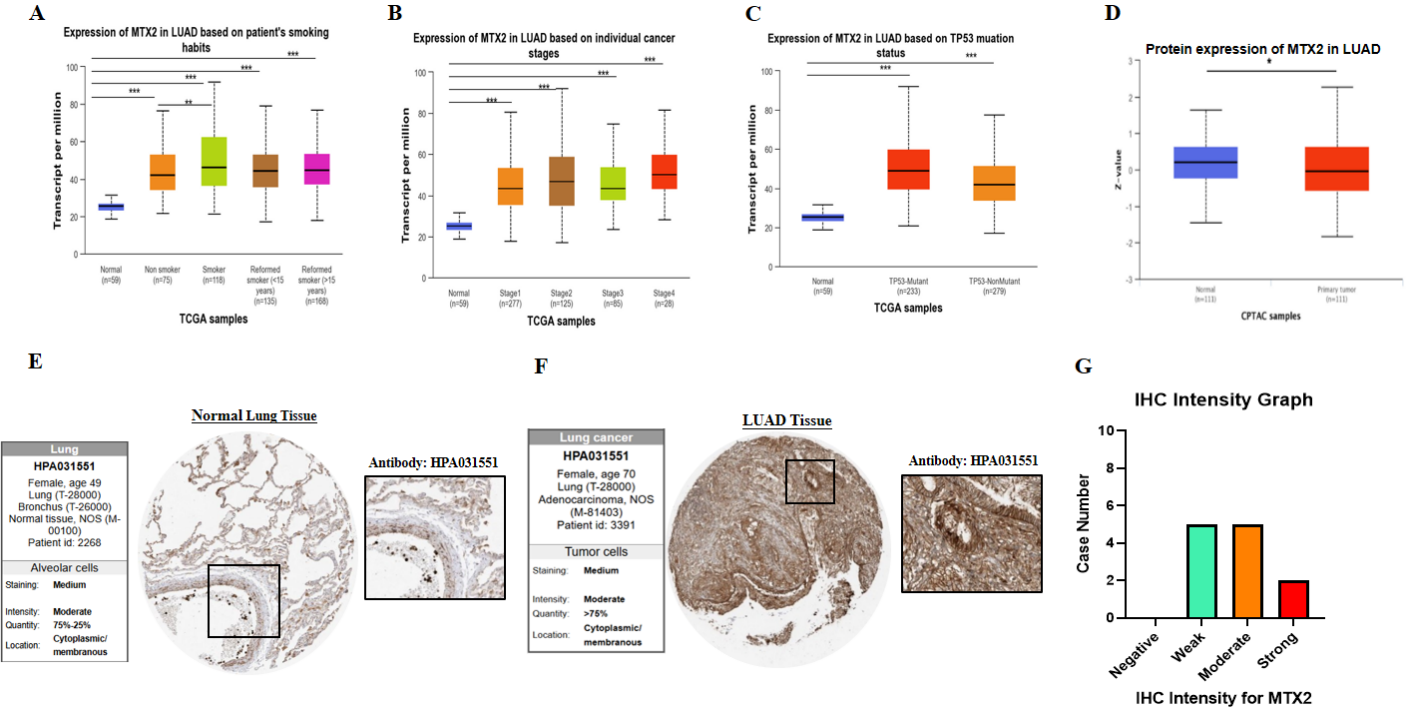
**Expression and analysis of MTX2 in lung adenocarcinoma (LUAD) patients. A:** mRNA levels between different patients' smoking habits and cancer stages. **B:** mRNA levels in different cancer stages. **C:** mRNA levels in the TP53 mutant. **D:** Protein level of MTX2 from CPTAC data by a *t*-test.** E, F:** Immunohistochemical (IHC) images illustrate the MTX2 staining intensity, with patient information provided for both normal and tumor samples from the Human Protein Atlas (HPA). **G:** Bar charts quantifying IHC staining intensities in LUAD specimens, enabling a comparative analysis. (* p < 0.05; ** p < 0.01; *** p < 0.001)

Our research revealed the differential expression of MTX2 across various cancer types, indicating consistent upregulation in a significant number of cancers compared to normal tissues. Furthermore, it was observed that MTX2 expression in LUAD varied in association with a patient's smoking behavior according statistical comparisons in supplementary Table S1 (Fig. 4A), specific cancer stages according to statistical comparisons in supplementary Table S2 (Fig. 4B), and an analysis of the TP53 mutation status in relation to MTX2 expression also revealed various levels according to statistical comparisons in supplementary Table S3 (Fig. 4C). Subsequently, we conducted a *t*-test using CPTAC data to assess MTX2 protein levels, which revealed decreased expression in LUAD compared to normal tissues (*p* = 5.567319e-01) (Fig. 4D). Immunohistochemical (IH) images from the HPA confirmed elevated MTX2 protein levels, aligning with the mRNA differential expression analysis (Fig. 4E, F). Notably, LUAD patient samples showed various MTX2 expression levels, highlighting its potential role in tumor progression. An analysis of antibody intensity data revealed strong staining in two of 12 samples, moderate staining in five, weak staining in five, and no negative staining (Fig. 4G). As part of this study, we created a heatmap to show the distribution of DNA methylation sites in MTX2 in LUAD (Supplementary Fig. S3C). We identified eight methylated CpG sites, with one site showing high expression levels. Among these, cg14504697 had the highest level of DNA methylation.

### Exploring pathways and disease associations through an enrichment analysis of MTX2 in LUAD

**Figure 5:**
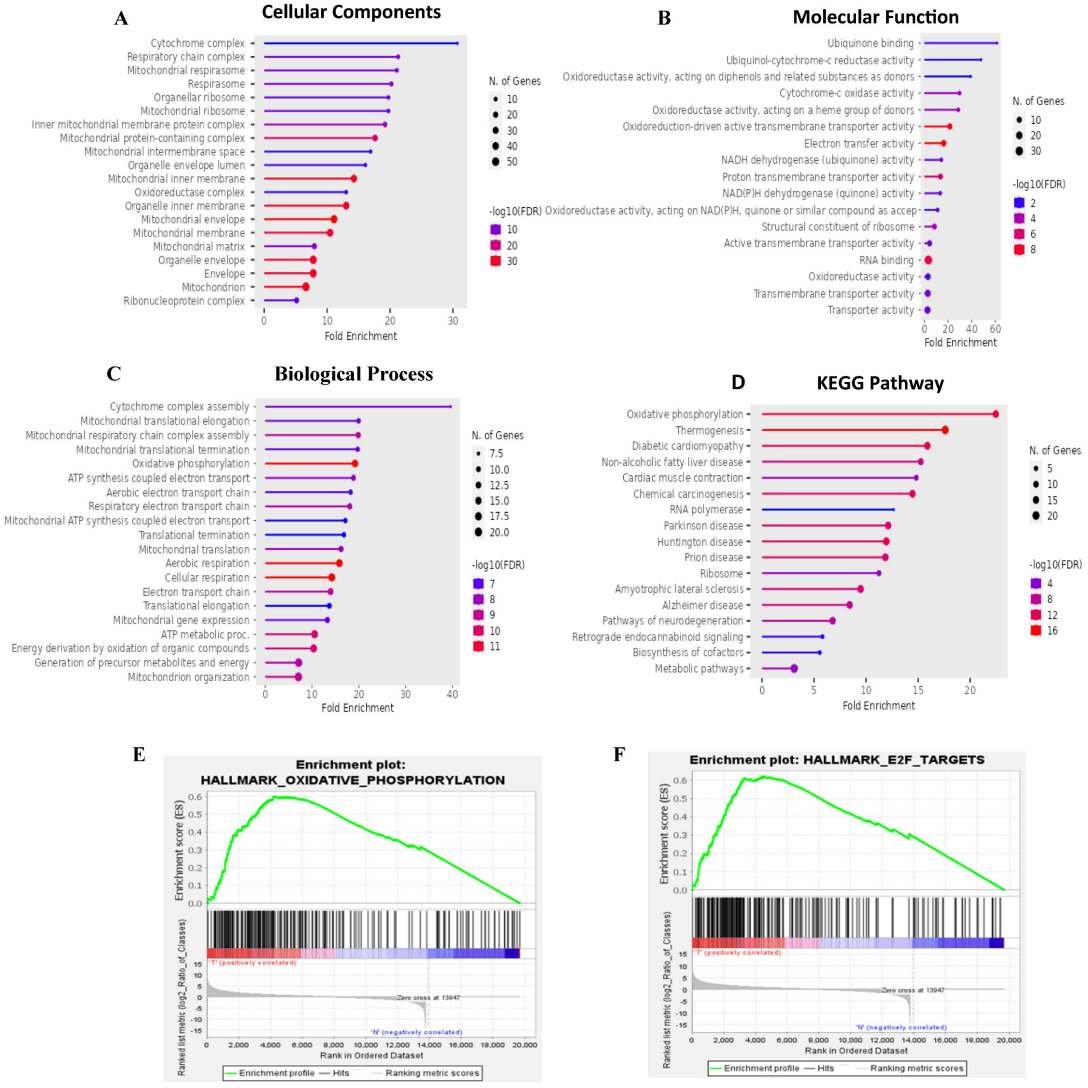
** Functional and pathway enrichment analyses of MTX2 (Gene Ontology (GO), Kyoto Encyclopedia of Genes and Genomes (KEGG), and Gene Set Enrichment Analysis (GSEA).** This figure presents results of functional and pathway enrichment analyses for MTX2. **A-C:** The top enriched categories from a GO analysis, focusing on cellular components (A), molecular functions (B), and biological processes (C). **D:** KEGG pathway analysis, revealing a strong association with the “oxidative phosphorylation” pathway. **E:** Enrichment plot for the Hallmark "oxidative phosphorylation" pathway. **F:** Findings for the Hallmark "E2F targets" pathway. GSEA identified MTX2-related pathways using selection criteria of a false detection rate (FDR) q < 0.25, normalized enrichment score (NES) > 1.3, and nominal *p* > 0.05.

Results of the GO enrichment study covered BPs, CCs, MFs, and KEGG pathways (Fig. 5A-D). The BP analysis revealed MTX2's involvement in cytochrome complex assembly and mitochondrial translational elongation. CC results showed associations with the cytochrome complex and organellar small ribosomal subunit. For MFs, MTX2 was linked to ubiquinone binding and ubiquinol-cytochrome-c reductase activity. The KEGG analysis further highlighted its roles in oxidative phosphorylation (OXPHOS) and thermogenesis. To characterize potential functions of the *MTX2* gene, a GSEA was performed on DEGs between MTX2-high and MTX2-low patients in each cancer type. In total, 510 gene sets were found which revealed the Hallmark Pathway analysis of *MTX2* with E2transcription factor (E2F) targets (NES 1.8745251, *p* = 0.0) (Fig. 5E) and OXPHOS (NES = 1.8173399, *p* = 0.0) (Fig. 5F). E2Fs are important for controlling cell cycle progression and DNA replication. When these factors are dysregulated, it has significant effects on LUAD, a type of non-small cell lung cancer (NSCLC)^63^. Abnormal E2F activity in LUAD may result in uncontrolled cell division, override cell cycle checkpoints, and hinder DNA damage responses, which can lead to tumor growth and genetic instability. Targeting E2F-associated pathways may delay cancer cell proliferation and induce cell death, making E2F dysregulation a potential biomarker for LUAD diagnosis and prognosis. MTX2's role in OXPHOS is also highly relevant to LUAD progression. This pathway, essential for ATP production in mitochondria, is often altered in cancer cells to meet increased energy demands. Elevated MTX2 expression in LUAD suggests that it may enhance mitochondrial function and OXPHOS, supporting the energy needs of proliferating tumor cells^64^, ^65^. MTX2's interaction with proteins involved in the mitochondrial structure, such as those in the MICOS complex, highlights its role in maintaining mitochondrial integrity and optimizing OXPHOS. Disruptions in mitochondrial function and CoQ10 metabolism can critically affect cancer cell survival and proliferation. Targeting the MTX2 and CoQ10 axis could present a novel therapeutic strategy to modulate mitochondrial function and suppress tumor growth in LUAD^66^, ^67^.

**Figure 6:**
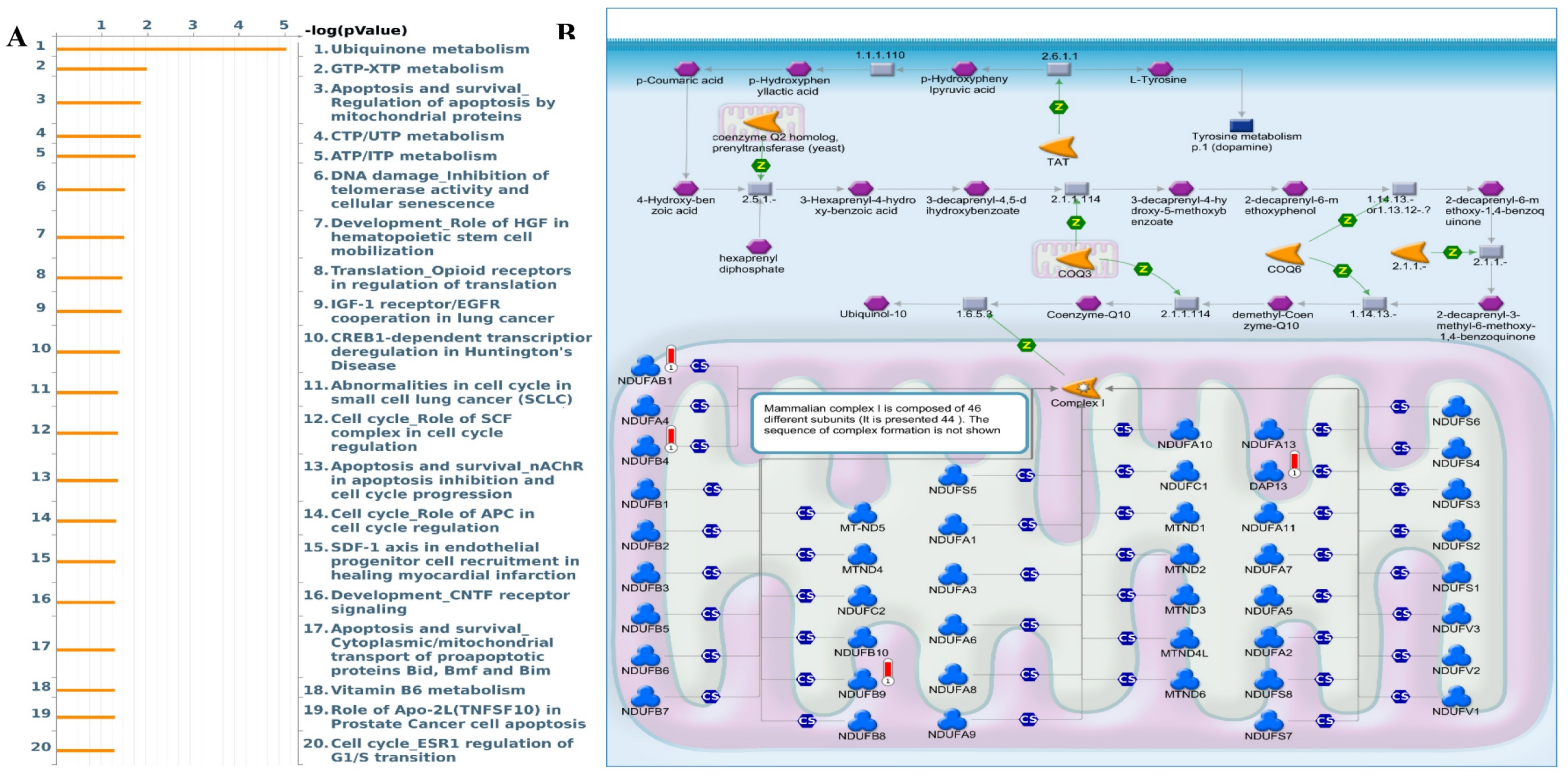
** MetaCore enrichment pathway analysis. A:** A MetaCore pathway enrichment analysis was conducted for *MTX2* co-expressed genes in lung adenocarcinoma (LUAD), revealing potential pathways involving these genes ranked by their log *p* values.** B:** The "ubiquinone metabolism pathway" is highlighted, with symbols representing proteins and arrows indicating protein interactions (green for activation and red for inhibition). Thermometer-like histograms visually representing microarray gene expressions, with blue indicating downregulation and red indicating upregulation.

Interestingly, the epithelial-to-mesenchymal transition (EMT) showed negative correlations with MTX2. MTX2 has a high expression and plays roles in cancer genes such as DNA repair (NES=1.4591016, *p*=0.005), fatty acid metabolism (NES=1.4519013, *p*=0.007), MTORC1 signaling (NES=1.2563931, *p*=0.043), P53 (NES=1.7069312, *p*=0.0), and MYC targeting of V2 (NES=1.7783564, *p*=0.0) (Supplementary Fig. S4)^63^. Multiple pathways and networks associated with metabolism were identified (Fig. 6A), including "ubiquinone metabolism" (Fig. 6B) and "GTP-XTP metabolism" (Supplementary Fig. S5). Detailed information on additional pathways and network-related data is provided in Supplementary Table S4. The conversion of coenzyme Q10 (CoQ10) to ubiquinol-10 is a key process in the mitochondrial respiratory chain which is essential for cellular energy production. CoQ10, in its oxidized form (ubiquinone-10), is first reduced to its semiquinone form (ubisemiquinone-10) by coenzyme Q-cytochrome c reductase (complex III), using electrons from ubiquinol-10 oxidation which produces superoxide (O2•-) as a byproduct. Semiquinone is then fully reduced to ubiquinol-10 by complex III via electrons from cytochrome c. Ubiquinol-10 facilitates electron transfer in the mitochondrial respiratory chain, driving ATP production and acting as a crucial antioxidant against oxidative damage. Notably, Hallmark ubiquinone showed significantly elevated expression in LUAD cells compared to normal cells, highlighting potential metabolic reprogramming in tumor progression.

### Immune expression analysis of MTX2 in LUAD

Tumor-infiltrating lymphocytes (TILs) have garnered significant attention in cancer immunotherapy for their capacity to migrate from the bloodstream into solid tumors and exert tumoricidal effects^68^. TIMER data indicated that the MTX2 gene is associated with immune system functions. Further analysis showed that there is a negative correlation between MTX2 and macrophages (Rho = -0.224, p = 4.91e-07), while no significant correlations were observed with other immune cells, such as CD8+ T cells, B cells, CD4+ T cells, neutrophils, macrophages, and dendritic cells. Additional details are provided in Supplementary Fig. S6. Moreover, analysis using the TISIDB database revealed a correlation between MTX2 mRNA expression and tumor-infiltrating lymphocytes (TILs). Notably, immature dendritic cells and CD56 dim cells were positively correlated with MTX2 expression, as shown in Supplementary Fig. S7.

### Single-cell analysis of MTX2 in LUAD

**Figure 7:**
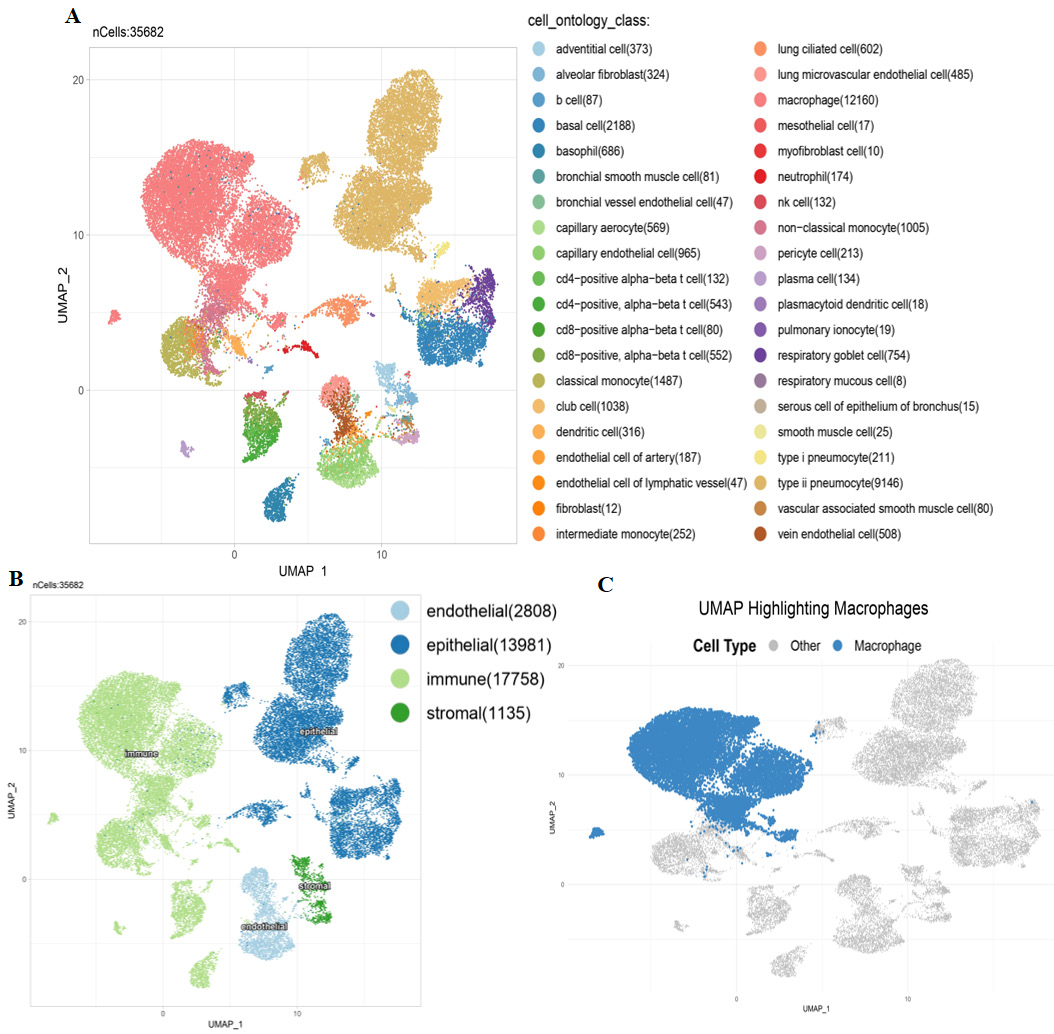
** Single-cell analysis:** Several key visualizations of an examination of MTX2 features in the lung adenocarcinoma (LUAD) tumor microenvironment (TME).** A:** First, single-cell cluster visualization using UMAP illustrates distinct types of TME cells, including immune cells. MTX2 expression in different cell types in **B:** UMAP plot visualizing the grouping of lung tissue cells according to their compartments: endothelial, epithelial, immunological, and stromal. **C:** Exclusive focus on macrophages within the immune compartment, using a UMAP plot to illustrate their distribution among other cell types.

To further investigate the TME in lung tissues, we performed an scRNA-Seq analysis on a dataset derived from the Human Protein Atlas (HPA). The scRNA-Seq analysis revealed a diverse range of cell types within the TME. Using UMAP clustering, we identified distinct populations, including alveolar epithelial cells, macrophages, and endothelial cells (Fig. 7A). MTX2 expression was notably higher in alveolar epithelial cells and specific subsets of macrophages, as shown by violin plots (Supplementary Fig. S8A). This suggests that MTX2 could be crucial for maintaining the metabolic or functional state of these cells. The heatmap analysis further supported this by highlighting different expression patterns of *MTX2* and its potential co-regulated genes, especially in epithelial and immune cell compartments. This indicates that MTX2 might be involved in processes like mitochondrial function, oxidative stress response, or immune modulation (Supplementary Fig. S8B). The UMAP overlay showed a spatial enrichment of MTX2 expression in macrophages and epithelial cells (Fig. 7B), emphasizing its importance in specific cell types. Further analysis of macrophages revealed two distinct populations: high and low MTX2 expressions, showcasing the diversity within macrophages. This indicates that MTX2 could potentially be involved in macrophage polarization or functional specialization, processes crucial for immune regulation and tissue homeostasis (Fig. 7C). This finding aligns with previous studies that highlighted the role of macrophages in regulating cellular metabolism and immune responses in cancer. Specifically, tumor-associated macrophages (TAMs) are known to influence the metabolic reprogramming of cancer cells, including the modulation of ubiquinone metabolism and oxidative phosphorylation (OXPHOS) pathways^69^, ^70^. The differentiation, polarization, and capability of macrophages to build a strong response towards cancer cells are regulated through metabolism. Mitochondria were reported to be dysfunctional in cancer. After the Warburg effect, mitochondria are considered to be involved in cancer cells as they are associated with mutations of mitochondrial (mt)DNA, nuclear (n)DNA, and the production of reactive oxygen species that regulate transcription factors to induce proliferation of cancer cells^71^.

The single-cell UMAP revealed elevated expression of MTX2 in macrophages, suggesting a potential role in enhancing CoQ10 biosynthesis, a crucial component of the mitochondrial electron transport chain. While CoQ10 is primarily known for its antioxidant properties and role in mitochondrial function, emerging evidence indicates its potential antitumor effects by modulating oxidative stress and metabolic homeostasis^72^, ^73^. However, previous studies showed that in the TME, macrophage-driven CoQ10 production may instead support cancer cell survival by enhancing mitochondrial resilience and metabolic adaptability. This highlights the therapeutic potential of targeting the MTX2 and CoQ10 axis to disrupt the metabolic interplay between immune cells and tumor cells^74^.

### Protein-Protein Interaction (PPI) network analysis of MTX2 in LUAD

**Figure 8:**
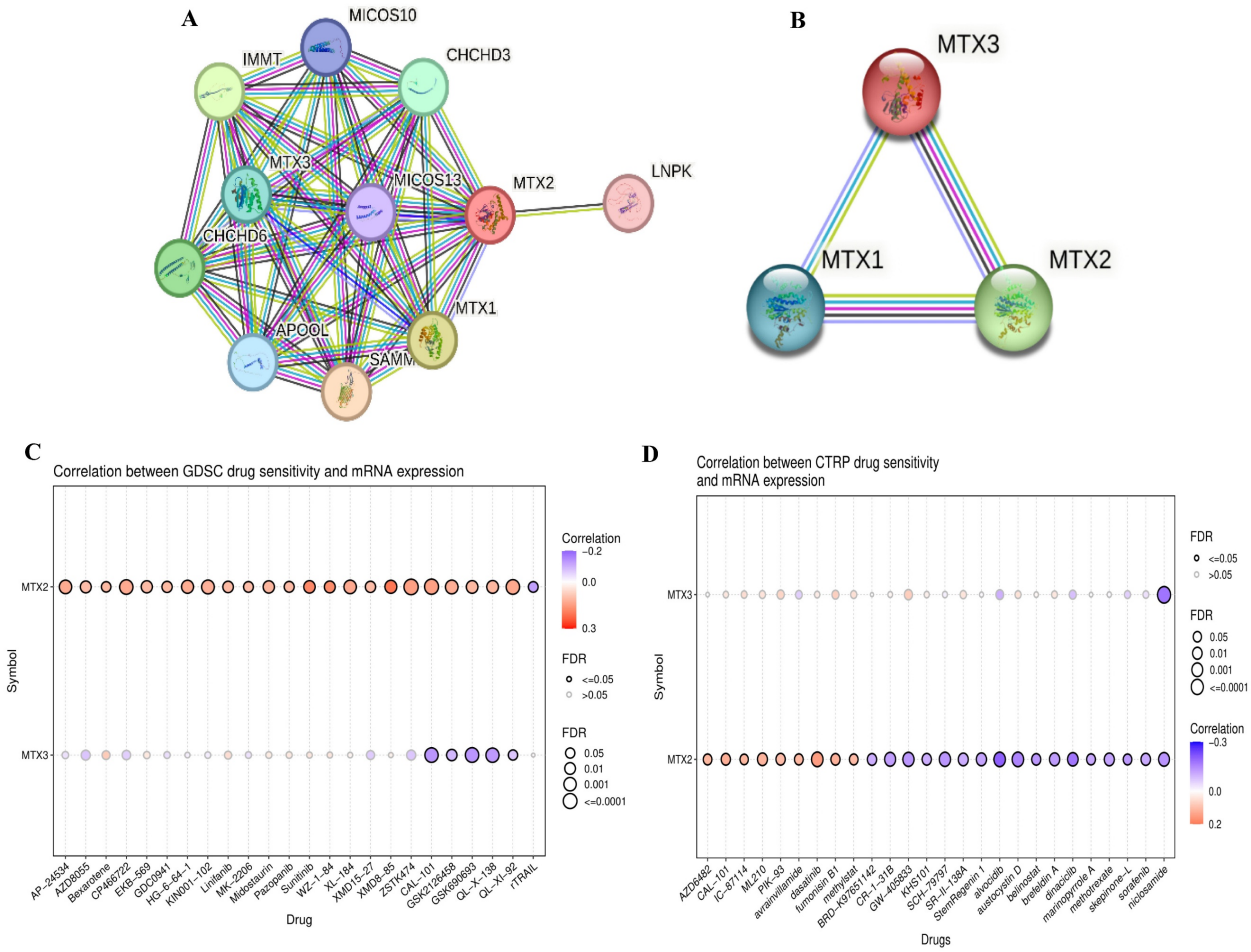
** Protein-Protein Interactions (PPIs) of MTX2 expression and drug analysis and prediction. A, B:** PPI network of MTX2 and **B** using data sourced from the STRING database. This network vividly illustrates interactions between MTX2 and other proteins, with highly interactive proteins depicted as hub nodes, shedding light on possible functional associations and partnerships involving MTX2. **C, D:** Study on MTX2's drug targets pinpointing ZSTK474 and CP466722 as key players in LUAD, targeting the PI3K pathway and CK2, while dasatinib from CTRP shows promise in disrupting LUAD signaling and is under clinical evaluation for its efficacy.

Using STRING, we analyzed PPIs of MTX2 and identified direct interactions with several key proteins, including MTX1, MTX3, MICOS10, MICOS13, TOMM6, IMMT, APOOL, CHCHD6, APOO, and CHCHD3. Notably, MTX2 also indirectly interacts with CoQ9, CoQ10B, and CoQ7 through IMMT. These interactions suggest that MTX2 is involved in critical mitochondrial functions and complexes (Fig. 8A, B). We also described combination scores of each protein with MTX2 (Supplementary Table S5). MTX1 and MTX3 are members of the metaxin family, playing critical roles in mitochondrial protein transport; MTX1 is essential for embryonic development and the import of proteins into mitochondria. The MICOS complex, composed of MICOS10, MICOS13, IMMT (mitofilin), CHCHD3, CHCHD6, APOOL, and APOO, is vital for maintaining the mitochondrial structure and function. IMMT and CHCHD3 are particularly important for junction formation and the organization of mitochondrial inner membranes. TOMM6 is a component of the translocase of the outer mitochondrial membrane (TOM) complex, which plays a critical role in the import of proteins into mitochondria. Furthermore, enzymes such as CoQ9, CoQ10B, and CoQ7 are essential for the biosynthesis of ubiquinone (CoQ10), a crucial element of the mitochondrial electron transport chain that supports OXPHOS by facilitating efficient electron transfer and ATP synthesis. The interactions of MTX2 with proteins like CHCHD3 and IMMT, which are part of the MICOS complex, highlight its involvement in preserving mitochondrial structure and function.^75^. These interactions imply that MTX2 is pivotal for maintaining mitochondrial integrity and cellular energy metabolism, both of which are essential in cancer progression and prognosis. In summary, our protein-protein interaction (PPI) and pathway analyses identified MTX2 as a key regulator of mitochondrial function, particularly in ubiquinone metabolism and oxidative phosphorylation (OXPHOS). These findings enhance our understanding of the molecular mechanisms through which MTX2 may impact LUAD progression and underscore its potential as a therapeutic target.

### Pharmacological target networks of MTX2 in LUAD

After conducting a comprehensive study of MTX2, an analysis was performed on drug target networks of MTX2 using the GSCA dataset to check the targets. The analysis revealed that the major pharmacological targets of MTX2 in GDSC were ZSTK474 and CP466722 (Fig. 8C). Research indicates that ZSTK474 impedes cell proliferation by causing cell cycle arrest in the G_1_ phase. Additionally, ZSTK474 suppresses the secretion of vascular endothelial growth factor (VEGF) and matrix metalloproteinases (MMPs), thereby inhibiting cell migration, invasion, and adhesion^76^. ZSTK474 represents an investigational drug with a focus on targeting the phosphatidylinositol-3-kinase (PI3K) pathway in lung cancer^77^. Its primary objective is to inhibit this pathway, thereby reducing cancer cell growth, triggering apoptosis, hindering angiogenesis, improving treatment sensitivity, and counteracting resistance mechanisms. CP466722, on the other hand, is an experimental drug designed to target protein kinase in cancer^78^. Its purpose is to inhibit ATM kinase, leading to suppression of cell proliferation, promotion of apoptosis, disruption of signaling pathways, enhancement of treatment response, and hindrance of metastasis. This drug was shown to play a pivotal role in targeting ATM kinase in cisplatin-resistant lung cancer cells. By inhibiting ATM, CP466722 induces the mesenchymal-to-epithelial transition, characterized by decreased expression of EMT-related genes, and significantly reduces cell invasion. It also downregulates programmed death ligand-1 (PD-L1) expression and impairs the Janus kinase 1/2 (JAK1/2)-signal transduction and activator of transcription 3 (STAT3) signaling pathway, which are key factors in tumor immune evasion and metastasis^79^. In the context of the Cancer Therapeutics Response Portal (CTRP; Fig. 8D), the major pharmacological target of MTX2 was identified as dasatinib. Dasatinib is a tyrosine kinase inhibitor drug that primarily targets proteins involved in cell signaling. In LUAD, its main objective is to disrupt abnormal signaling pathways responsible for cancer cell growth, spread, and survival. Specifically, dasatinib inhibits Src-family kinases (SFKs) and the BCR-ABL fusion protein^80^. Its potential roles in LUAD include curbing cancer cell growth, inhibiting invasion and metastasis, influencing epidermal growth factor receptor (EGFR)-mutant cancers, and synergizing with other therapies. Ongoing clinical trials are focused on determining its effectiveness in various LUAD scenarios.

### Biological behaviors of MTX2 in LUAD cancer cells

**Figure 9:**
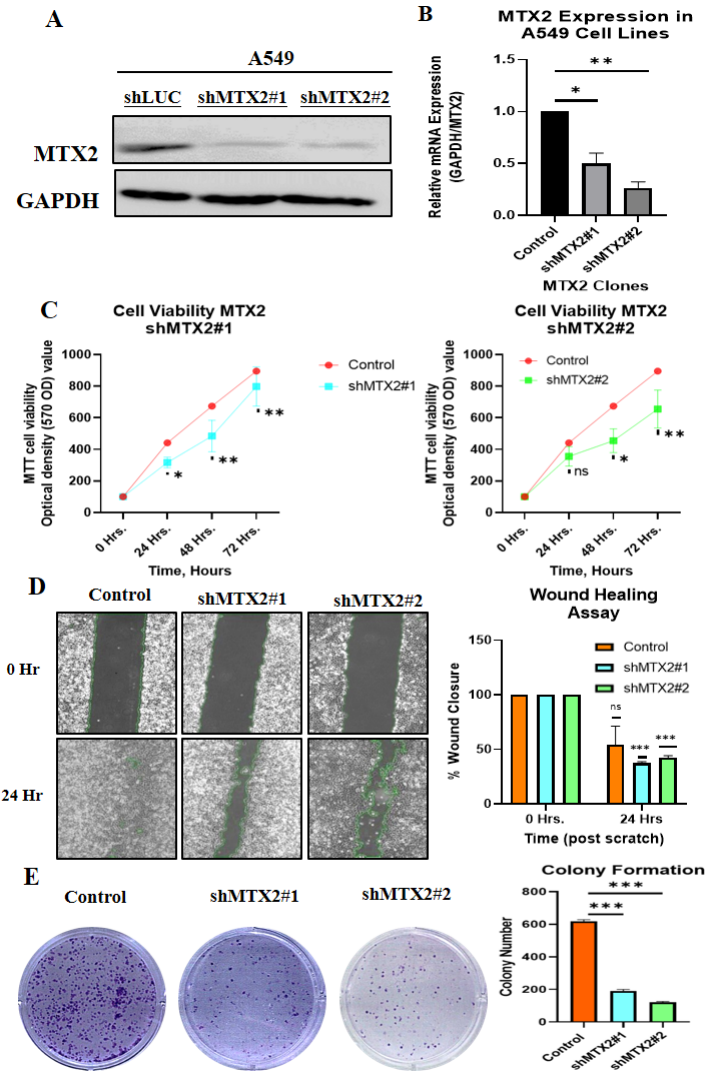
** Knockdown efficacy of MTX2 in the A549 cell line. A, B:** Western blot analysis of MTX2 protein expression levels and RT-qPCR analysis of *MTX2* mRNA expression levels after lentiviral transduction in the A549 cell line. **C:** MTT assay showing that knockdown of MTX2 inhibited A549 cell proliferation. **D:** Wound-healing assay showing that knockdown of MTX2 promoted A549 cell migration. **E:** Representative images of the colony-formation assay in the knockdown of MTX2 groups with histogram quantification. Significance (* p < 0.05; ** p < 0.01; *** p < 0.001)

This study aimed to validate and investigate the role of MTX2 in LUAD by manipulating its expression levels in A549 cancer cells. Using homemade lentiviral transfection, MTX2 was knocked-down in A549 cells, resulting in the construction of shMTX2#1 and shMTX2#2 cell lines. The efficacy of MTX2-knockdown (KD) was confirmed through RT-qPCR and Western blot analyses (Fig. 9A, B), demonstrating a significant downregulation of mRNA and protein expression levels of MTX2 compared to the control (shLuc). SDs were determined by experiments performed in triplicate. Further analysis using MetaCore revealed that MTX2 plays a role in the ubiquinone metabolism pathway, as demonstrated by gene expression profiling with CoQ10A and CoQ10B primers (Supplementary Fig. S9), indicating its potential regulatory influence on mitochondrial function (Supplementary Fig. S10A, B). CoQ10 is known for its role in mitochondrial bioenergetics and its antioxidant properties. Elevated CoQ10 levels enhance mitochondrial function and protect against oxidative damage, aligning with MTX2's observed effects in LUAD cells^81^, ^82^. Recent studies suggested that CoQ10 influences cancer cell metabolism by modulating expressions of genes involved in proliferation and survival. Elevated CoQ10 levels support mitochondrial function, energy production, and oxidative stress resistance. MTX2-KD enhances CoQ10 biosynthesis, potentially influencing mitochondrial function and cellular metabolism. These findings suggest that targeting the MTX2/CoQ10 axis may represent a viable therapeutic strategy for LUAD and other cancers^83^-^85^.

### Functional assays for MTX2 in LUAD cells

The effect of MTX2-KD on cell proliferation was evaluated using an MTT assay at 0, 24, 48, and 72 hours of incubation. The results revealed a substantial reduction in cell proliferation in MTX2-KD A549 cells compared to shLuc control cells, underscoring the essential role of MTX2 in the proliferative capacity of LUAD cells (Fig. 9C). A wound-healing assay was performed to assess the impact of MTX2 on the motility of A549 cells. The data revealed a significant reduction in cell motility following MTX2-KD, as evidenced by diminished scratch healing rates. Knockdown of MTX2 inhibited the invasion and migration abilities of A549 cells in vitro (Fig. 9D). Furthermore, colony-formation assays showed that silencing MTX2 significantly reduced both the number and size of colonies formed by A549 cells, suggesting that MTX2 contributes to the proliferative and self-renewal capabilities of LUAD cells (Fig. 9E).

## Discussion

This study establishes *MTX2* as a pivotal gene in various cancers, particularly in LUAD, by demonstrating its correlations with poor patient prognoses and tumor progression. A bioinformatics analysis revealed differential expression and DNA methylation patterns of MTX2 in LUAD, suggesting its potential as a prognostic biomarker. Our wet lab validation further confirmed that knockdown of MTX2 expression reduced levels of key oncogenic markers and altered the immune cell infiltration profile, aligning with the pathways predicted by MetaCore. Univariate and multivariate Cox analyses revealed a significant correlation between MTX2 expression and advanced disease stages (stages III and IV with respective hazard ratios of 3.0 and 2.38). Functional assays demonstrated that MTX2 promotes LUAD cell proliferation and inhibits apoptosis, implicating it in cellular stress responses and mitochondrial function. The GO analysis identified significant enrichment of genes co-expressed with *MTX2* in CCs such as the cytochrome complex and respiratory chain complex, highlighting its role in mitochondrial processes essential for tumor progression. KEGG and MetaCore pathway analyses further confirmed MTX2's involvement in OXPHOS and ubiquinone metabolism, which are crucial for sustaining cancer cell metabolism and survival.

A single-cell analysis provided evidence of a strong association between MTX2 expression and macrophages within LUAD tissues. A positive correlation between MTX2 and macrophage suggested a role in immunomodulation. Understanding this relationship could provide new insights into MTX2's impact on tumor progression and metabolic pathways. This was supported by increased *CoQ10* mRNA levels in MTX2-KD A549 cells, implying that MTX2 may enhance CoQ10 biosynthesis and promote mitochondrial bioenergetics. These findings align with a broader understanding that TAMs can influence several metabolic pathways, including ubiquinone metabolism and OXPHOS, contributing to cancer cell survival and proliferation^86^, ^87^. Our experiments provide empirical support for the hypothesis that MTX2 influences the CoQ10 pathway through the observation of a significant upregulation of *CoQ10* mRNA levels in MTX2-KD A549 cells. This suggests that MTX2 may enhance CoQ10 biosynthesis, thereby promoting mitochondrial bioenergetics and protecting LUAD cells from oxidative stress. Conversely, MTX2-KD resulted in significant reductions in cell proliferation and colony formation were observed, accompanied by an increase in apoptosis.

MTX2's role in mitochondria and its involvement in the tumor immune system make it a potential therapeutic target. Inhibiting MTX2 could disrupt mitochondrial function, decrease tumor cell proliferation, and might enhance the efficacy of treatments or help overcome resistance, such as to cisplatin. This offers a potential solution to the challenge of platinum resistance in lung cancer. Developing small-molecule inhibitors or monoclonal antibodies against MTX2 could be a promising approach. Existing compounds might be repurposed, or new ones could be designed based on the structure and function of MTX2. Combination therapies involving MTX2-targeted treatments with other drugs that target mitochondrial function or OXPHOS could provide a synergistic effect. Preclinical and clinical investigations are required to assess MTX2 inhibitors, in order to evaluate the safety and efficacy of MTX2-targeted therapeutic strategies^30^, ^88^. Preliminary bioinformatics analyses suggested that pharmacological agents such as ZSTK474 (a PI3K inhibitor) and CP466722 (a protein kinase CK2 inhibitor) may influence MTX2-associated pathways, revealing opportunities for drug repurposing or combination therapies.

**Figure 10:**
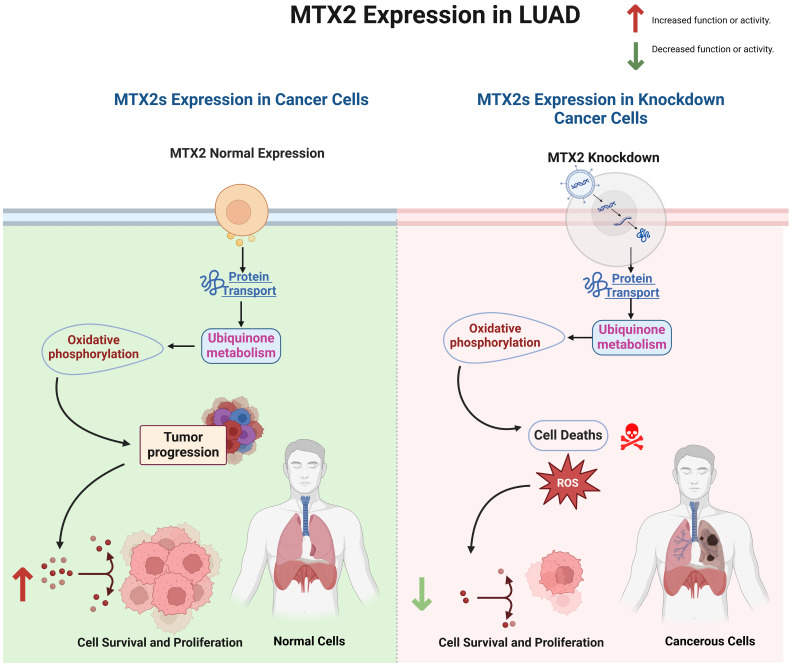
Interpreting MTX2-associated functional alterations in lung adenocarcinoma.

MTX2 might be a potential biomarker, as patients with high MTX2 expression might benefit more from MTX2-targeted therapies. MTX2 has emerged as a promising biomarker and therapeutic target in LUAD, but it exhibits low cancer specificity, underscoring its involvement in broader oncogenic processes. This suggests that MTX2 is integral to cellular stress responses and mitochondrial functions that are critical for cancer cell survival and proliferation in diverse malignancies. Future research should delve into mitochondrial dynamics, OXPHOS, and CoQ10 biosynthesis, Additionally, exploring MTX2's interactions with immune cells, particularly TAMs in the TME could discover new therapies. Lastly, evaluating MTX2's specific roles and interactions in other cancer types could provide a more-comprehensive understanding of its oncogenic potential and improve prognostic accuracy, ultimately paving the way for improved patient outcomes (Fig. 10).

## Conclusions

In conclusion, our study highlights the crucial role of the MTX2 gene in LUAD, emphasizing its potential as both a valuable biomarker and a therapeutic target. The increased expression of MTX2 in LUAD tissues was found to correlate with poor patient outcomes, further supporting its relevance in disease progression and prognosis. Functional assays revealed that MTX2- knock down suppressed tumor growth, as evidenced by reduced colony formation. Additionally, wound-healing assays indicated a slower migration rate in MTX2-knock down LUAD cells, underscoring its oncologic properties. Additionally, our findings revealed a significant association between MTX2 and key metabolic processes, including OXPHOS and ubiquinone metabolism, as well as a notable correlation with macrophage populations within the TME. These interactions indicate a complex network in which MTX2 modulates both metabolic pathways and immune responses, potentially affecting tumor progression and therapeutic efficacy. Elucidating these interactions could uncover novel therapeutic targets and strategies to enhance patient outcomes.

## Supplementary Material

Supplementary figures and tables.

## Figures and Tables

**Table 1:** Basic characteristics of MTX2, including its recognized HGNC (HUGO Gene Nomenclature Committee) and gene ID, and acronyms

Gene symbol	HGNC ID	Gene ID	Aliases	Ensemble	Description	Location on chromosome	Function
MTX1	7504	4580	MTXN; MTX; mitochondrial outer membrane imports complex protein 1, Metaxin 1	ENSG00000173171	Metaxin 1: Predicted to be involved in mitochondrion organization. Located in the SAM complex. Part of the MIB complex.	1q22	Involved in the transport of proteins into the mitochondrion. Essential for embryonic development (By similarity).
MTX2	7506	10651	The mitochondrial outer membrane imports complex protein 2, Metaxin 2	ENSG00000128654	Metaxin 2: Involved in mitochondrial transport. Located in the SAM complex and nucleolus. Part of the MIB complex.	2q31.1	Involved in the transport of proteins into the mitochondrion.
MTX3	24812	345778	Metaxin-3	ENSG00000177034	Metaxin 3: Predicted to be involved in mitochondrion organization. Part of the MIB complex and SAM complex.	5q14.1	Could function in the transport of proteins into the mitochondrion.

## Abbreviations

BP: biological process
CC: cellular component
cDNA: complementary DNA
E2F: E2 transcription factor
FDR: false detection rate
FPS: first-progression survival
GEPIA: Gene Expression Profiling Interactive Analysis
GSEA: gene set enrichment analysis
KD: knockdown
KEGG: Kyoto Encyclopedia of Genes and Genomes
MF: molecular function
MTT: 3-(4, 5-dimethylthiazolyl-2)-2, 5-diphenyltetrazolium bromide
NES: normalized enrichment score
NSCLC: non-small cell lung carcinoma
OS: overall survival
OXPHOS: oxidative phosphorylation
PPI: protein-protein interaction
PPS: post-progression survival
RT-qPCR: reverse-transcription quantitative polymerase chain reaction
STRING: Search Tool for the Retrieval of Interacting Genes/Proteins
TAMs: tumor-associated macrophages
TCGA: The Cancer Genome Atlas
