# Supplementary material for: Integration of multi-omics and single-cell transcriptome reveals mitochondrial outer membrane protein-2 (MTX-2) as a prognostic biomarker and characterizes ubiquinone metabolism in lung adenocarcinoma
Source: J Cancer. 2025 Apr 13;16(7):2401–20. doi: 10.7150/jca.106902 (PMC12036103; doi:10.7150/jca.106902)
Supplement: Supplementary file 1 — Supplementary figures and tables. [file jcav16p2401s1.pdf]

Supplementary Figures:

Fig. S1.

A

B

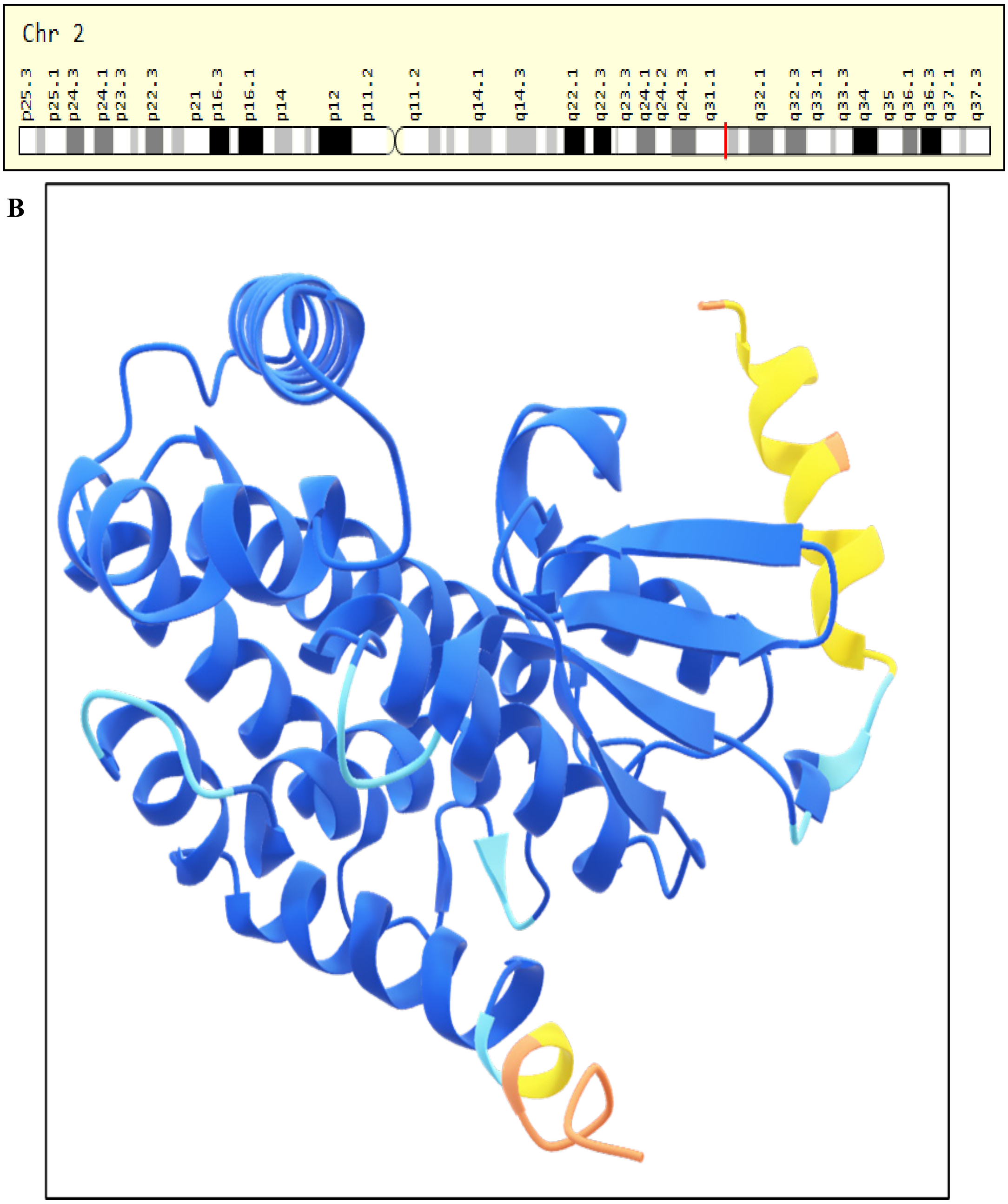

Supplementary Figure_S1: A*:* Depiction of the location of the *MTX2* gene on chromosome i.e., 2q31.1 with a size of 68,631 bases. It encodes mitochondrial outer membrane import complex protein 2. **B:** Depiction of a 263-aa-long protein with a weight of 29.763 kDa which is involved in the transport of proteins into mitochondria.

Fig. S2.

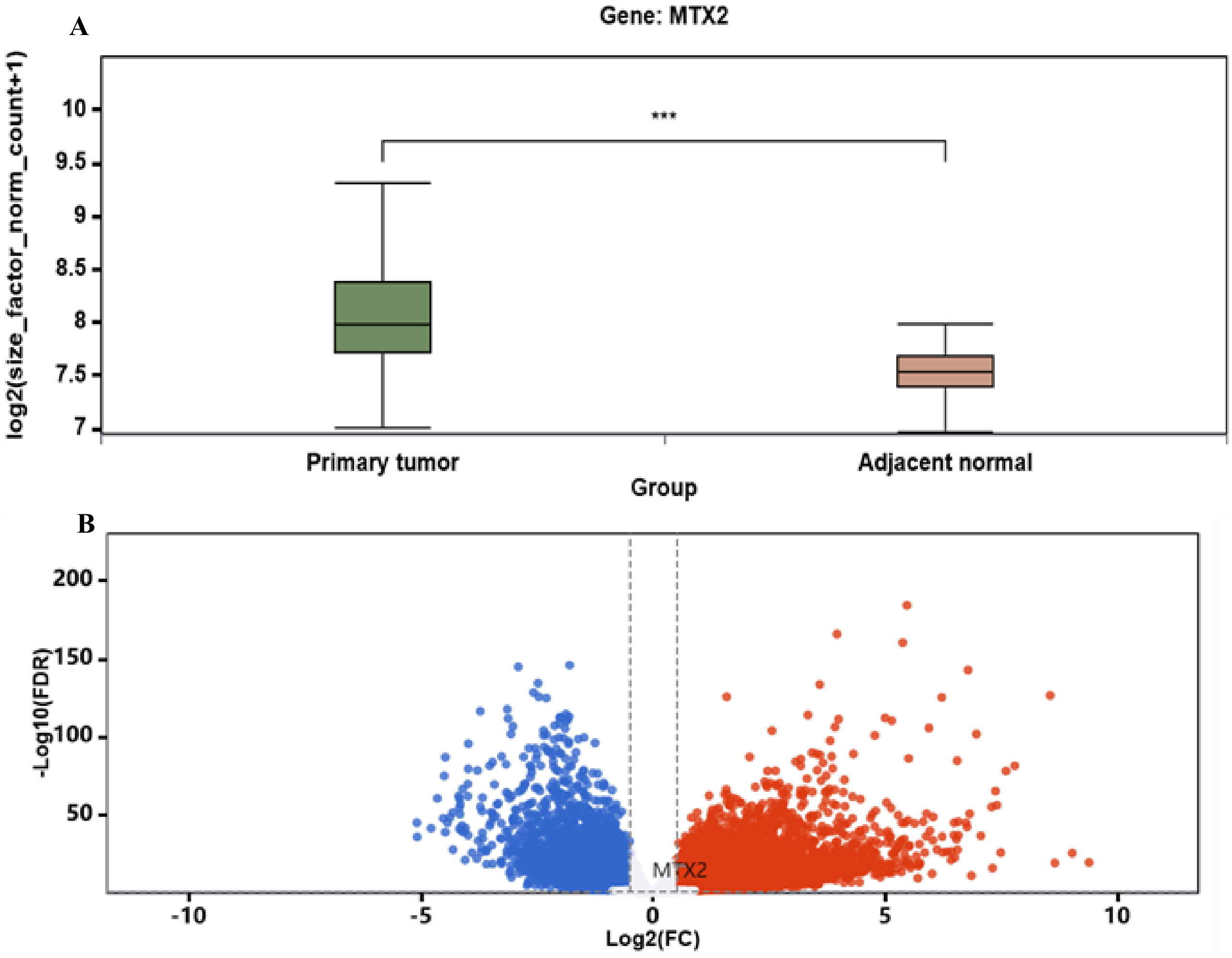

Supplementary Figure_S2: A, B: Differentially expressed gene (DEG) validation using a BioTuring Deseq2 analysis of data from SRP074349 (GSE81089), filtered for the adenocarcinoma histological type.

Fig. S3.

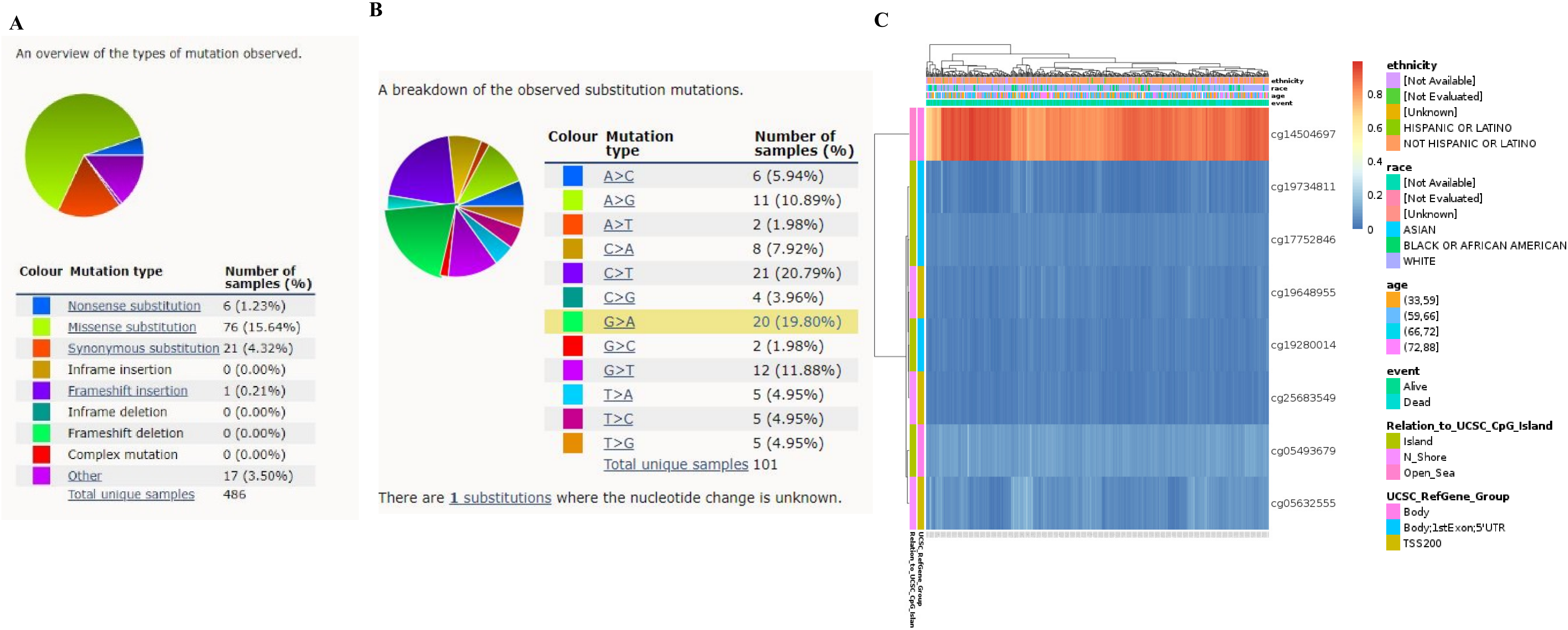

Supplementary Figure_S3: A, B: COSMIC dataset used to examine gene mutations in the context of lung adenocarcinoma (LUAD). The diagram displays various mutation types. **C:** Heatmap from the MethSurv platform, illustrating *MTX2* DNA methylation levels in LUAD. Blue bars indicate low expression, while red bars indicate high expression, with colored boxes denoting factors like ethnicity, race, age, events, UCSC CpG island relations, and UCSC RefGene groups. β-values (0–1) represent the methylation status, with cg14504697 showing the highest DNA methylation levels.

Fig. S4.

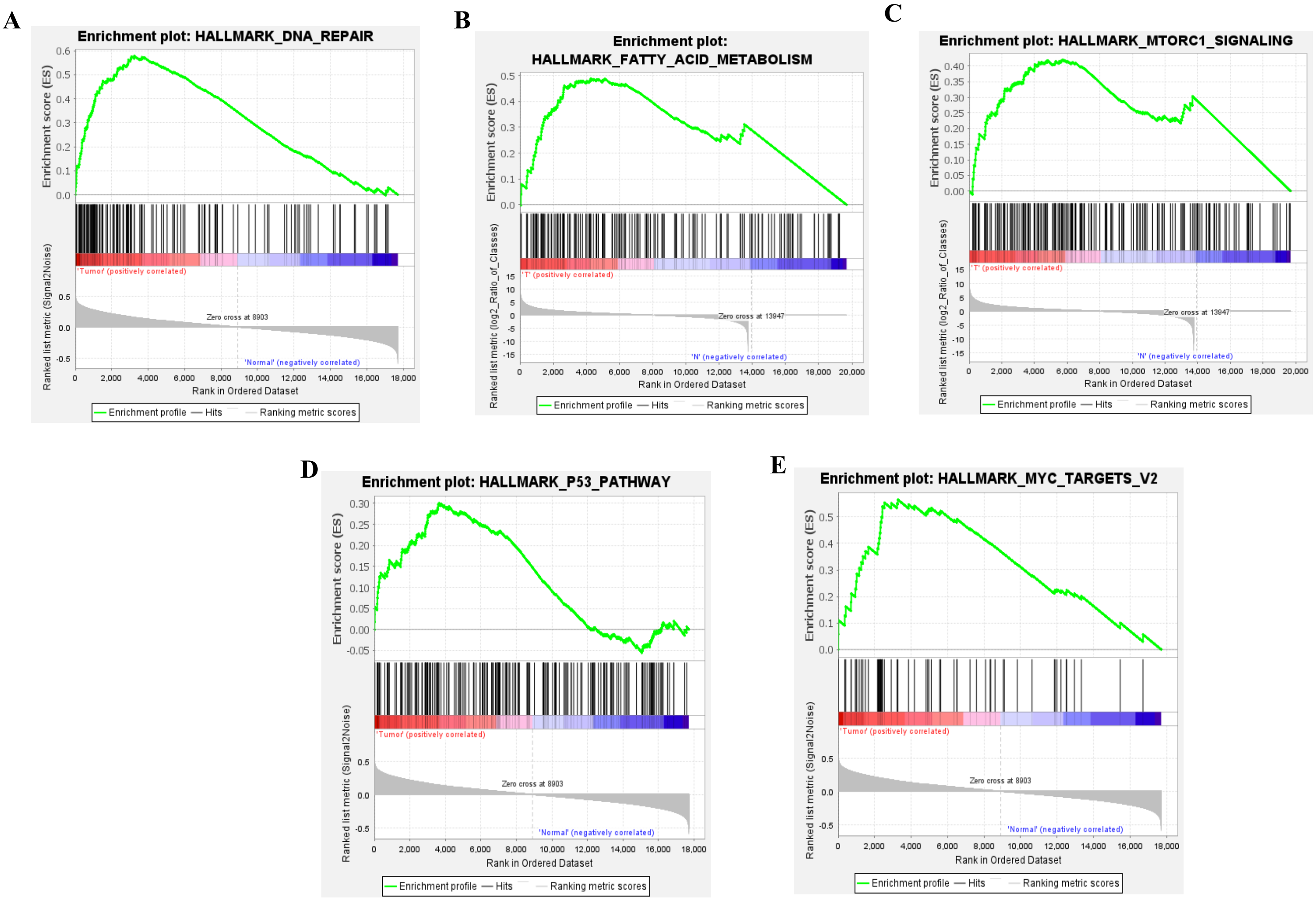

Supplementary Figure_S4: Gene set enrichment analysis (GSEA) results of MTX2 expression in lung adenocarcinoma (LUAD). **A-E:** GSEA identification of MTX2-related signaling pathways; these criteria included a false detection rate (FDR) q value of < 0.25, a normalized enrichment score (NES) of > 1.3, and a nominal *p* value of > 0.05.

Fig. S5.

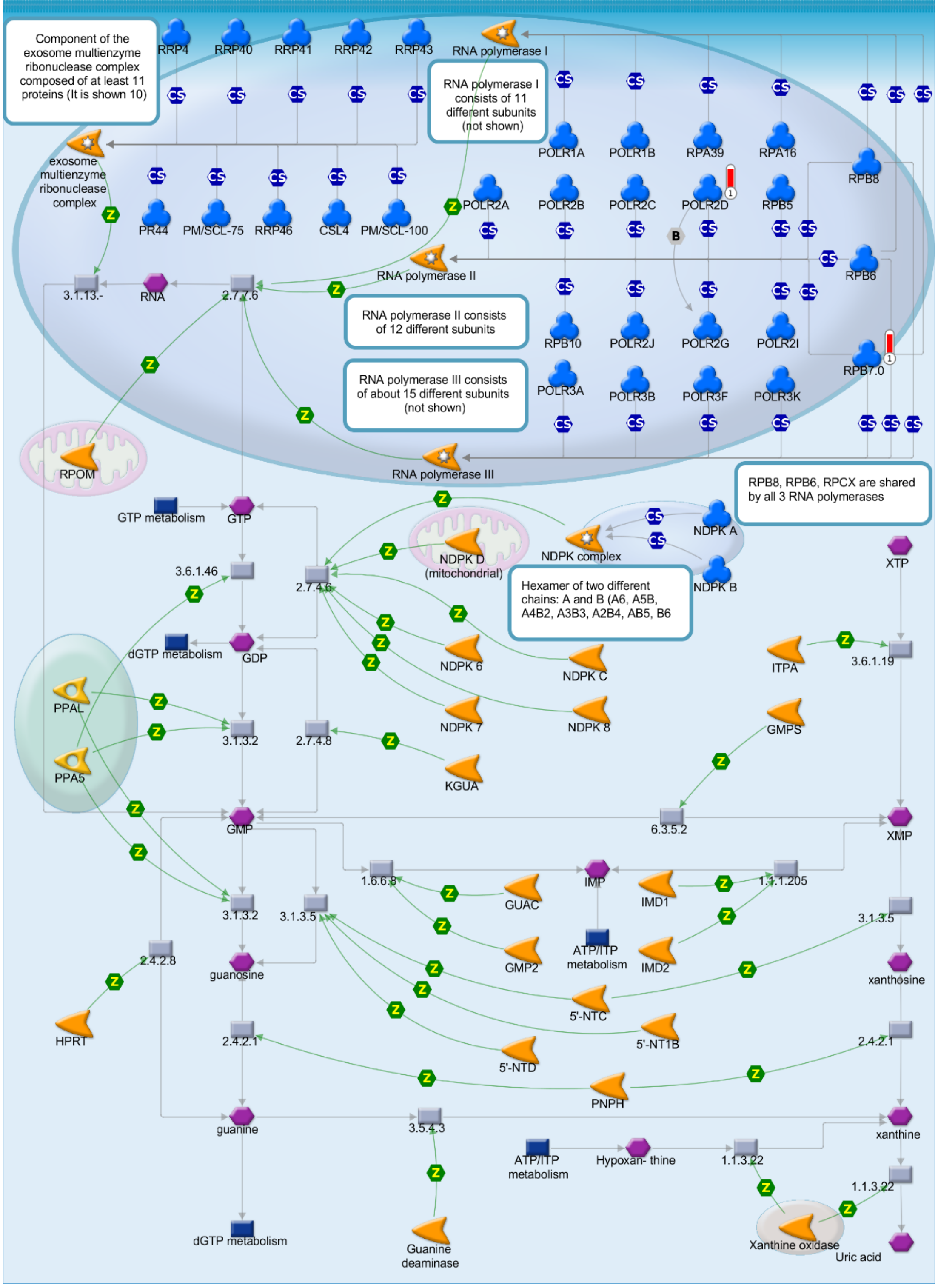

Supplementary Figure_S5: MetaCore enrichment pathway (GTP-XTP metabolism) analysis of genes co-expressed with *MTX2*.

Fig. S6.

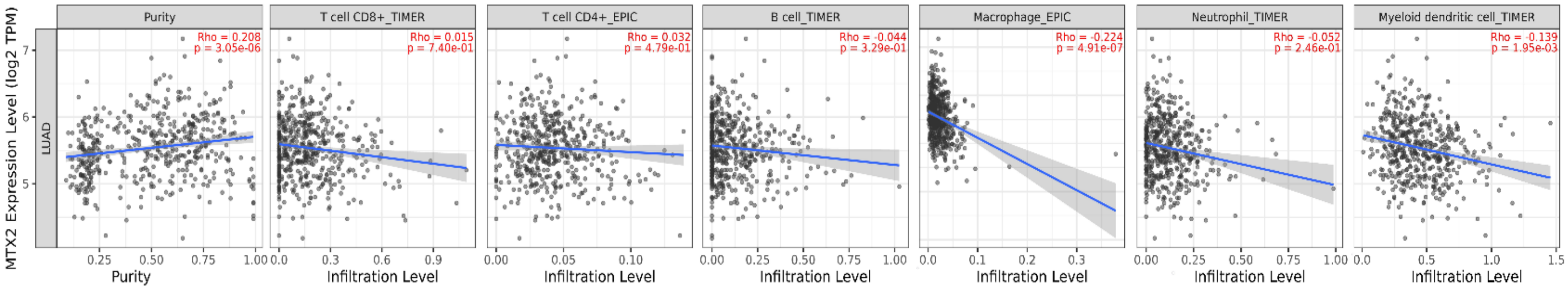

Supplementary Figure_S6: Associations between expression levels of the *MTX2* gene and various immune cell markers, including clusters of differentiation 8-positive ($CD8^+$) T cells, B cells, $CD4^+$ T cells, neutrophils, macrophages, and dendritic cells. Spearman correlations were employed to quantify relationships between the *MTX2* gene and immune cells, considering $p < 0.05$ as statistically significant.

Fig. S7.

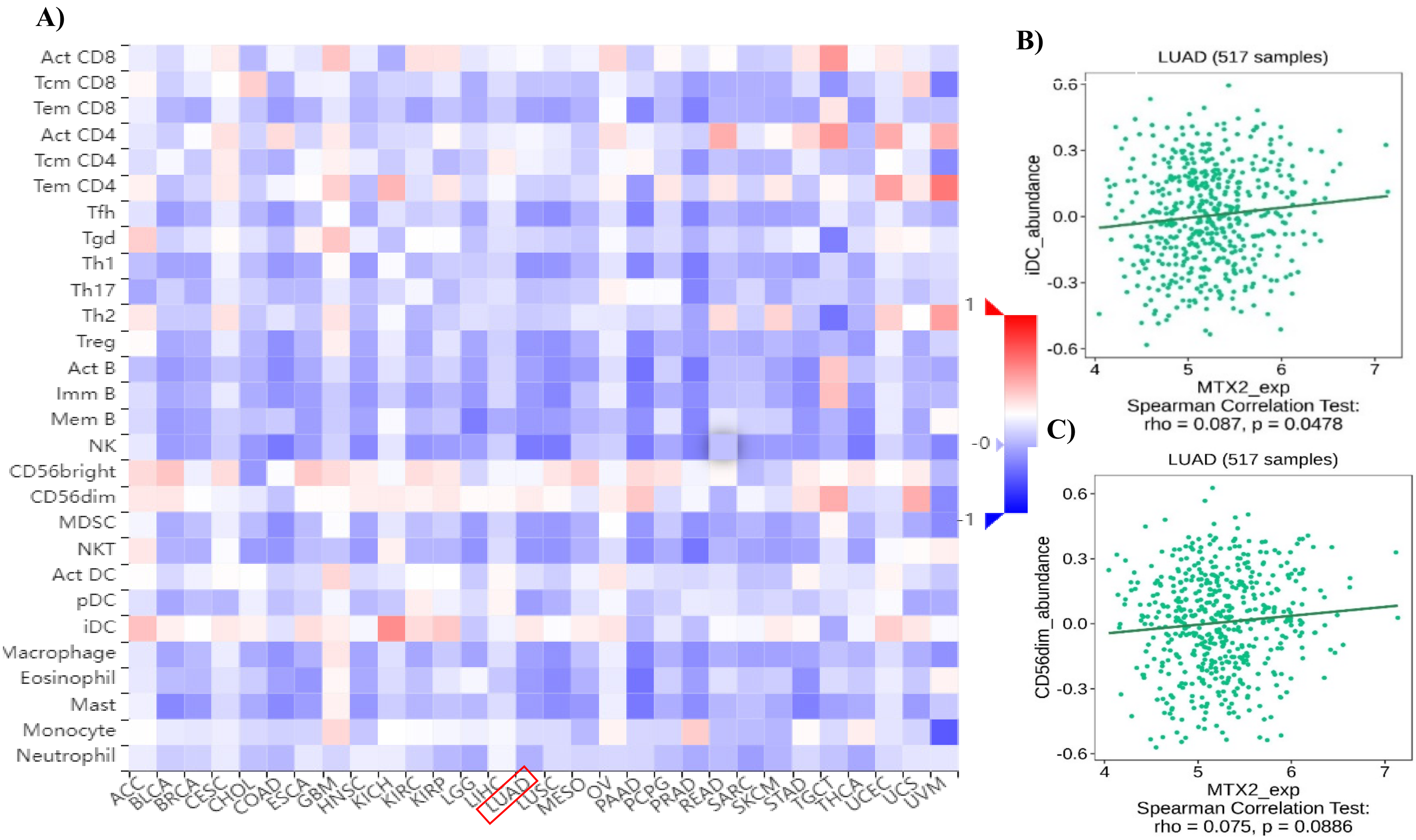

Supplementary Figure_S7: Analysis of TISIDB, which shows relationships between the abundance of tumor-infiltrating lymphocytes (TILs) and the expression, copy number, methylation, or mutation of the *MTX2* gene. Charoentong's study provides immune-related signatures for 28 TIL types of cancer. In each cancer type, the relative abundance of TILs was determined using a gene set variation analysis (GSVA) based on gene expression profiles. **A:** Which types of TILs might be regulated by the *MTX2* gene. **B:** Immature dendritic cells and CD56dim were highly expressed.

Fig. S8. A)

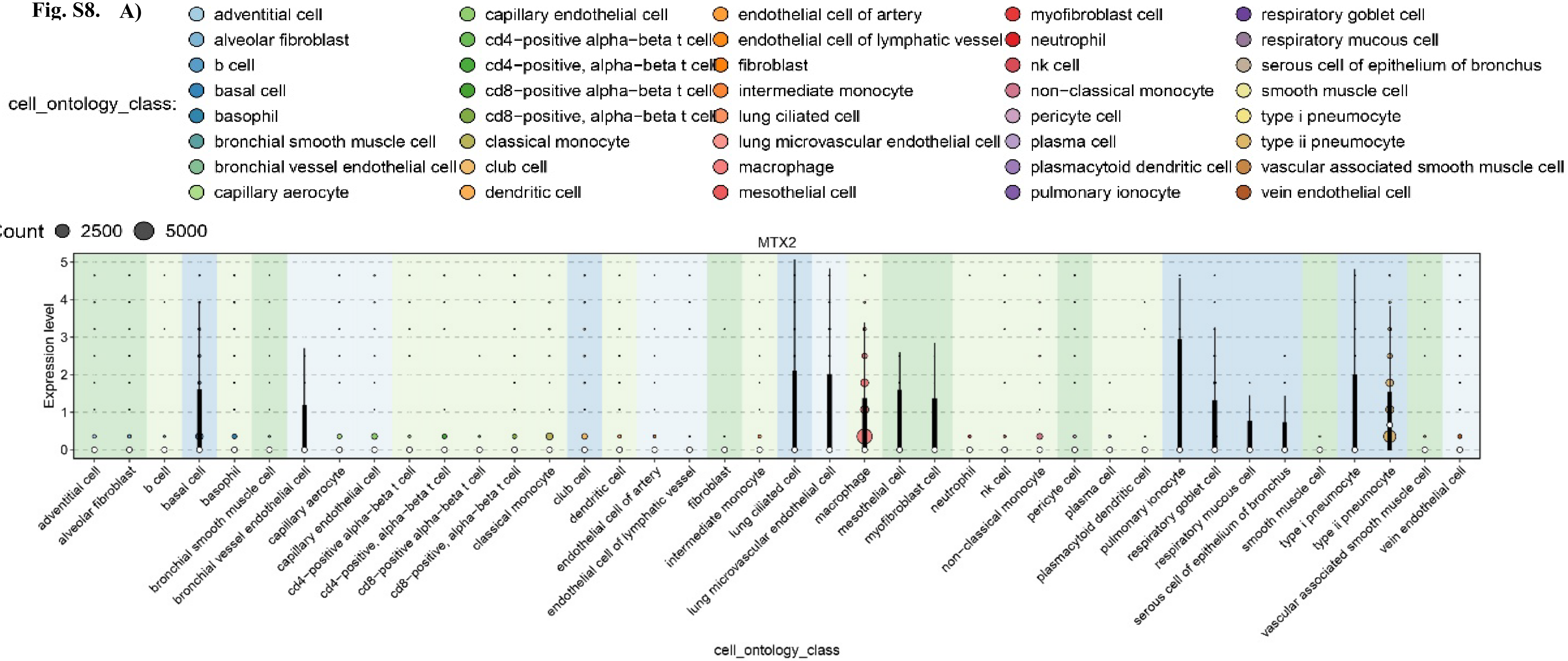
cell_ontology_class:
adventitial cell
alveolar fibroblast
b cell
basal cell
basophil
bronchial smooth muscle cell
bronchial vessel endothelial cell
capillary aerocyte
capillary endothelial cell
cd4−positive alpha−beta t cell
cd4−positive, alpha−beta t cell
cd8−positive alpha−beta t cell
cd8−positive, alpha−beta t cell
classical monocyte
club cell
dendritic cell
endothelial cell of artery
endothelial cell of lymphatic vessel
fibroblast
intermediate monocyte
lung ciliated cell
lung microvascular endothelial cell
macrophage
mesothelial cell
myofibroblast cell
neutrophil
nk cell
non−classical monocyte
pericyte cell
plasma cell
plasmacytoid dendritic cell
pulmonary ionocyte
respiratory goblet cell
respiratory mucous cell
serous cell of epithelium of bronchus
smooth muscle cell
type i pneumocyte
type ii pneumocyte
vascular associated smooth muscle cell
vein endothelial cell
Count 2500 5000
MTX2
Expression level
0
1
2
3
4
5
cell_ontology_class

B)

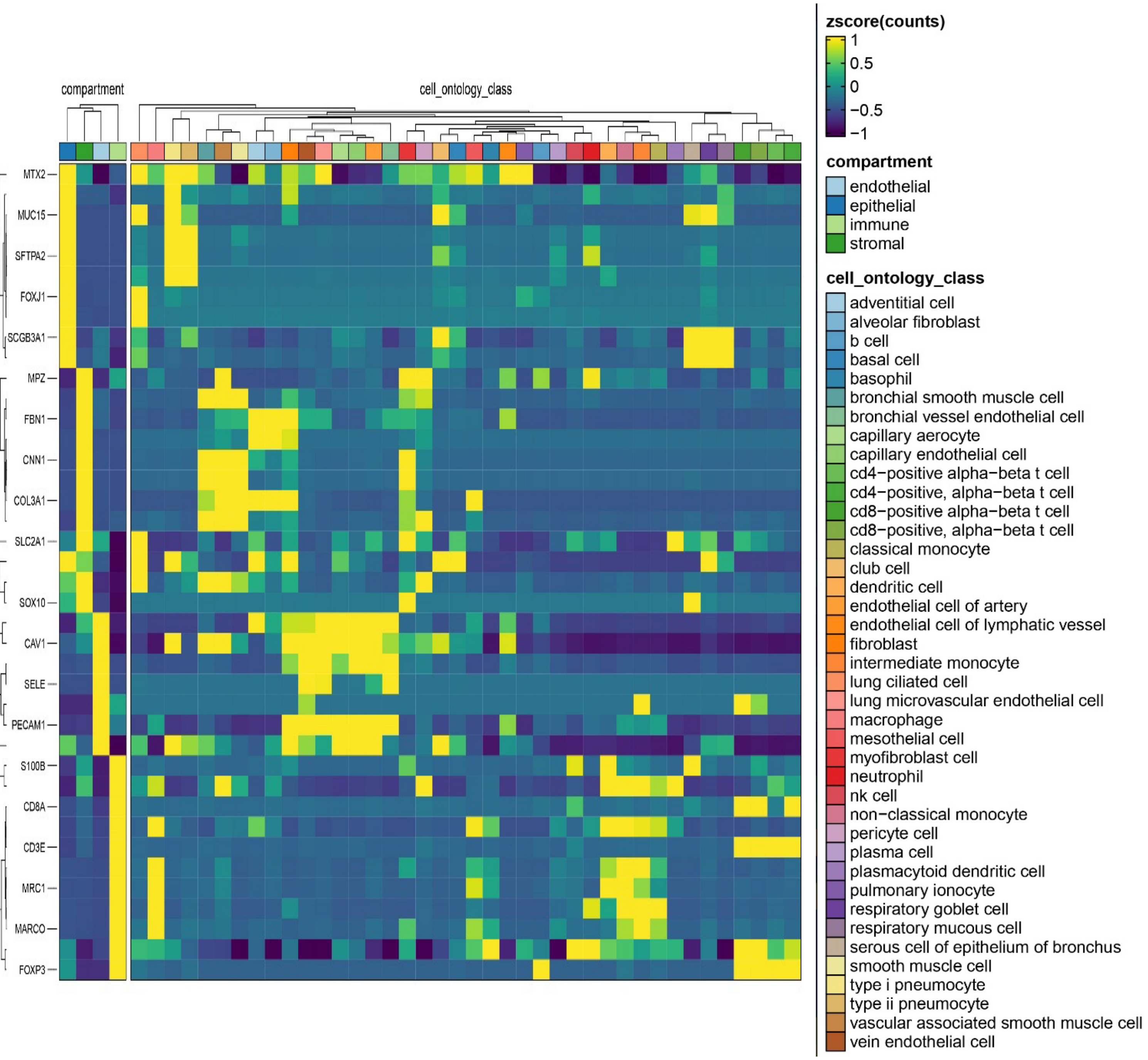

Supplementary Figure_S8: A: Violin plot representing MTX2 gene expression levels across diverse cell types within the lung microenvironment. The x-axis denotes different cell ontology classes, while the y-axis indicates expression levels. Colors correspond to distinct cell types, as detailed in the legend. **B:** Gene expression heatmap by cell type and compartment, showing the z-score normalized expression of key genes across four compartments: endothelial, epithelial, immunological, and stromal, along with their respective cell types.

Fig. S9.

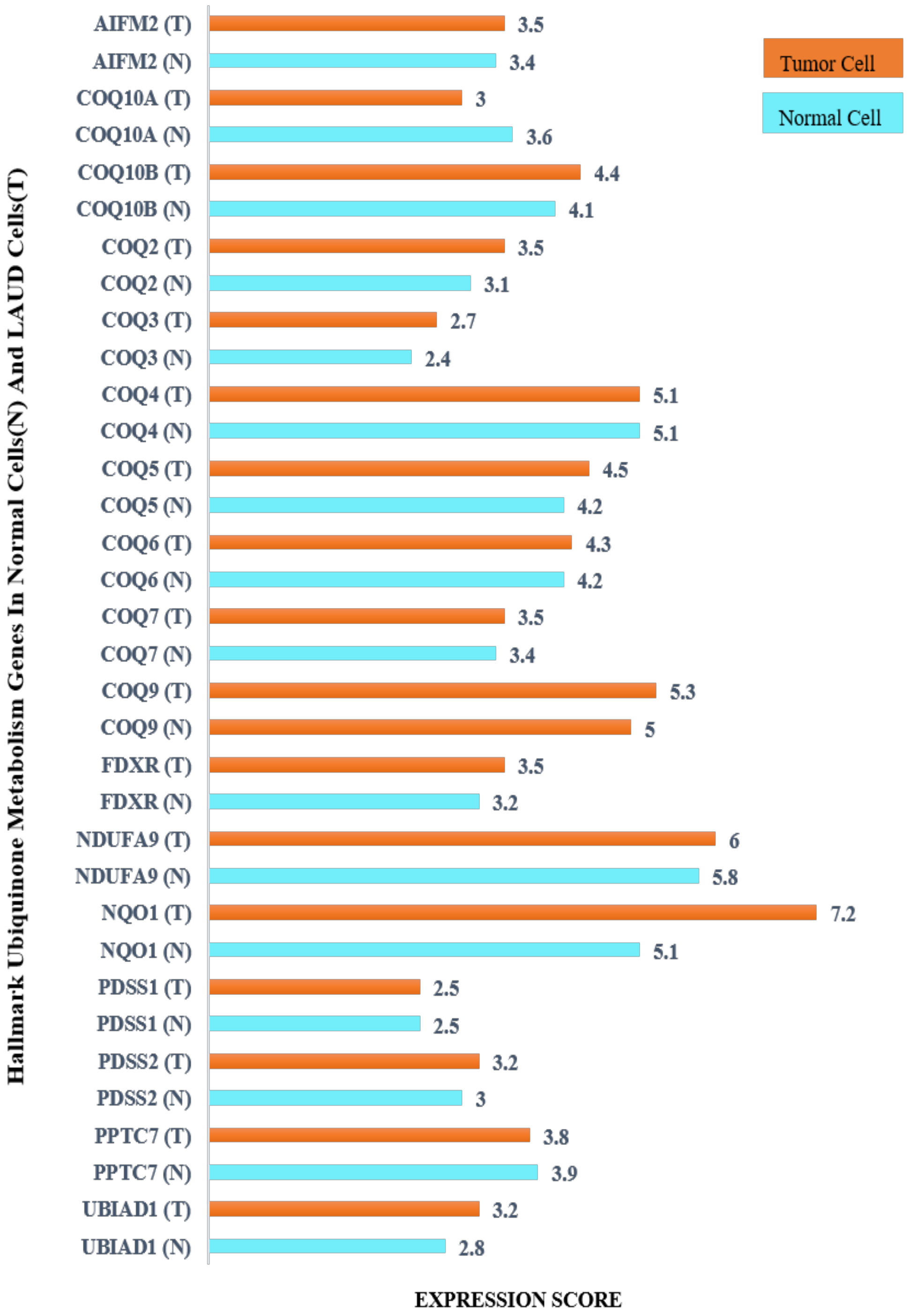

Supplementary Figure_S9: Expression levels of genes related to ubiquinone metabolism in lung adenocarcinoma (LUAD) cells compared to normal cells.

Fig. S10.

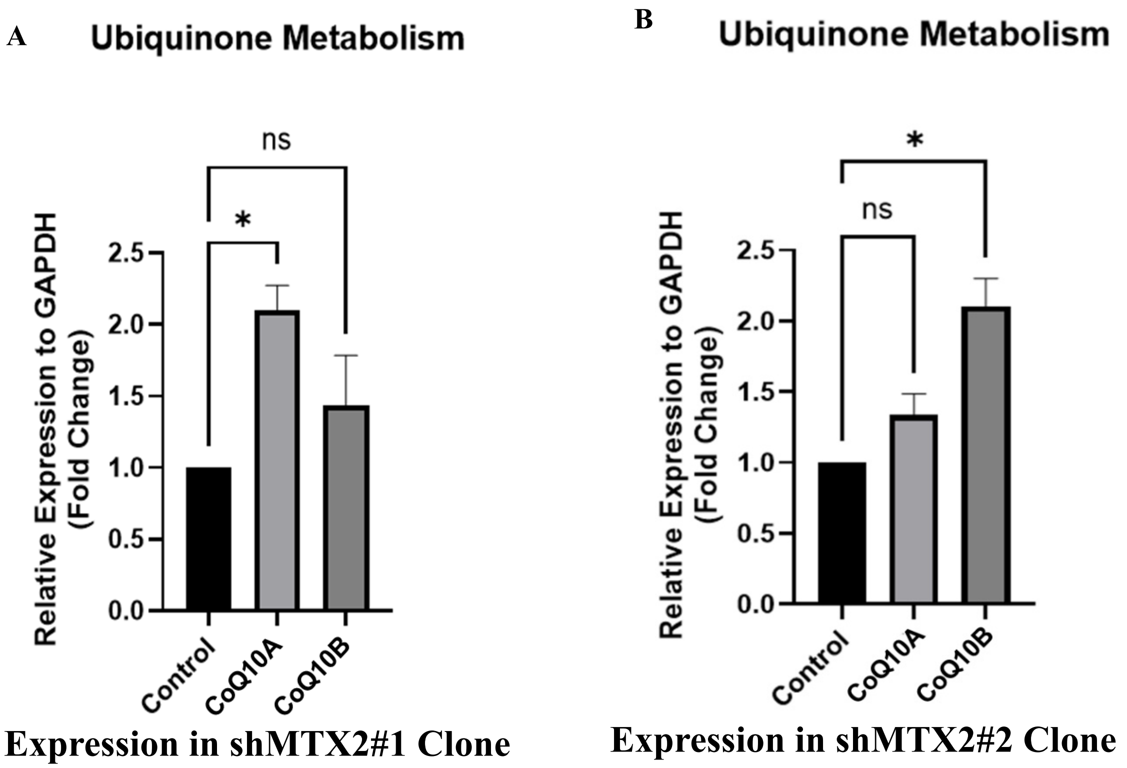

Supplementary Figure_S10: *CoQ10A* and *CoQ10B* mRNA levels. RT-qPCR analysis demonstrating increased mRNA expressions of *CoQ10A* and *CoQ10B*, key regulators of ubiquinone metabolism influenced by MTX2. Data were normalized using GAPDH, and statistical significance was assessed using an ANOVA, with error bars representing the standard deviation (SD) of triplicate determinations ($p < 0.05$, * $p < 0.01$).

Table Legends:

Table S1: Statistical comparison of the patients' smoking behaviors with the *MTX2* gene.

**Comparison**	**Statistical significance**
Normal-vs-Non-smoker	<1E-12
Normal-vs-Smoker	<1E-12
Normal-vs-Reformed smoker1	1.62447832963153E-12
Normal-vs-Reformed smoker2	<1E-12
Non-smoker-vs-Smoker	2.479500E-03
Non-smoker-vs-Reformed smoker1	1.653200E-01
Non-smoker-vs-Reformed smoker2	7.414100E-02
Smoker-vs-Reformed smoker1	6.082200E-02
Smoker-vs-Reformed smoker2	1.100030E-01
Reformed smoker1-vs-Reformed smoker2	6.508600E-01

Table S2: Statistical comparison of the individual cancer stages with the *MTX2* gene.

Comparison	Statistical significance
Normal-vs-Stage1	1.62447832963153E-12
Normal-vs-Stage2	<1E-12
Normal-vs-Stage3	<1E-12
Normal-vs-Stage4	4.25026680517249E-12
Stage1-vs-Stage2	3.973000E-01
Stage1-vs-Stage3	5.406400E-01
Stage1-vs-Stage4	1.448800E-01
Stage2-vs-Stage3	9.079000E-01
Stage2-vs-Stage4	3.535600E-01
Stage3-vs-Stage4	3.051000E-01

Table S3: Statistical comparison of the TP53 mutant status of the *MTX2* gene.

Comparison	Statistical significance
Normal-vs-TP53-Mutant	1.62436730732907E-12
Normal-vs-TP53-NonMutant	1.62436730732907E-12
TP53-Mutant-vs-TP53-NonMutant	7.35710004029499E-09

Table S4: Pathway analysis of genes co-expressed with *MTX2* from the MetaCore database (with $p < 0.05$ set as the cutoff value).

**S.No.**	**Maps**	**pValue**	**Network Objects from Active Data**
1	Ubiquinone metabolism	9 250E-06	NDUFB9, NDUFB4, DAP13, NDUFAB1
2	GTP-XTP metabolism	1 174E-02	POLR2D, RPB7.0
3	Apoptosis and survival_Regulation of apoptosis by mitochondrial proteins	1 602E-02	MFF, Smac/Diablo
4	CTP/UTP metabolism	1 631E-02	POLR2D, RPB7.0
5	ATP/ITP metabolism	2 120E-02	POLR2D, RPB7.0
6	DNA damage_Inhibition of telomerase activity and cellular senescence	3 613E-02	La protein
7	Development_Role of HGF in hematopoietic stem cell mobilization	3 791E-02	RHEB2

8	Translation_Opioid receptors in regulation of translation	4 144E-02	RHEB2
9	IGF-1 receptor/EGFR cooperation in lung cancer	4 321E-02	RHEB2
10	CREB1-dependent transcription deregulation in Huntington's Disease	4 673E-02	COX VIa-1
11	Abnormalities in cell cycle in small cell lung cancer (SCLC)	5 198E-02	CKS1
12	Cell cycle_Role of SCF complex in cell cycle regulation	5 198E-02	CKS1
13	Apoptosis and survival_nAChR in apoptosis inhibition and cell cycle progression	5 198E-02	RHEB2
14	Cell cycle_Role of APC in cell cycle regulation	5 721E-02	CKS1
15	SDF-1 axis in endothelial progenitor cell recruitment in healing myocardial infarction	5 895E-02	RHEB2
16	Development_CNTF receptor signaling	6 068E-02	RHEB2

17	Apoptosis and survival_Cytoplasmic/mitochondrial transport of proapoptotic proteins Bid, Bmf and Bim	6 068E-02	Smac/Diablo
18	Vitamin B6 metabolism	6 068E-02	Phospho2
19	Role of Apo-2L(TNFSF10) in Prostate Cancer cell apoptosis	6 068E-02	Smac/Diablo
20	Cell cycle_ESR1 regulation of G1/S transition	6 241E-02	CKS1

Table S5: Combination scores from the protein-protein interaction (PPI) analysis using STRING.

NODE 1	NODE 2	COMBINED SCORE
MTX2	MTX1	0.996
MTX2	IMMT	0.990
MTX2	CHCHD3	0.984
MTX2	MTX3	0.984
MTX2	MICOS10	0.950
MTX2	MICOS13	0.946
MTX2	APOO	0.939
MTX2	TOMM6	0.708
MTX2	TOMM20L	0.645
